# 3D heterotypic models of glioblastoma reveal the impact of microglia on cellular organization and the production of a distinct secretome

**DOI:** 10.1038/s41598-026-37395-0

**Published:** 2026-02-04

**Authors:** Clara García-Sáez, Josune Alonso-Marañón, Mikel García-Puga, Ane Rubio-Zulaika, Irati de Goñi-Garcia, Lorea Blázquez, Sandra Camarero-Espinosa

**Affiliations:** 1 BioSmarTE Lab, POLYMAT, University of Basque Country UPV/EHU, Av. de Tolosa, 72, Donostia-San Sebastián, 20018 Spain; 2 Neurosciences Area, Biogipuzkoa Health Research Institute, San Sebastian, 20014 Spain; 3 Neurosurgery department, Donostia University Hospital, San Sebastian, 20014 Spain; 4 Ikerbasque, Basque Foundation for Science, Euskadi Pl., 5, Bilbao, 48009 Spain; 5 Centro de Investigación Biomédica en Red de Enfermedades Neurodegenerativas (CIBERNED), Instituto de Salud Carlos III, Madrid, Spain

**Keywords:** Glioblastoma, Multicellular spheroids, Microglia, Patient-derived cells, Malignancy, Spatial arrangement, Cancer, Cell biology

## Abstract

Glioblastoma (GBM) is a deadly brain tumor with a very poor prognosis. Development of new therapeutics is hindered by the lack of appropriate preclinical models that reflect the complexity of the tumor microenvironment, especially the crucial role of microglia. In this study, we investigated the impact of microglia on GBM models using humanized 3D spheroids. Homotypic and heterotypic spheroids were created out of a GBM-derived cell line (DKMG) or patient-derived glioma stem cells (GB22-13), along with a microglia cell line (HMC3). Heterotypic glioma-HMC3 spheroids exhibited increased proliferation and greater drug resistance to chemotherapy drug Temozolomide compared with homotypic spheroids. Heterotypic spheroids also grew larger, developed multinucleated structures within 7 days, and had a greater invasive potential. Additionally, a distinct core-shell structure emerged in the heterotypic spheroids, with glioma cells concentrated in the core and a surrounding layer of microglia forming a protective shell that appeared to hinder drug penetration to the tumor core. Further, heterotypic cells were able to induce migration and polarization of peripheral blood monocytes (THP-1) towards M2 phenotypes, increasing immune evasion. These findings highlight the critical role of microglia in GBM development and progression, demonstrating their contribution to both reduced drug diffusion and increased tumor growth.

**Supplementary Information:**

The online version contains supplementary material available at 10.1038/s41598-026-37395-0.

## Introduction

Glioblastoma (GBM) is one of the most malignant and lethal brain cancers. Characterized by a poor prognosis, current treatments combine a highly invasive surgical resection, Tumor Treating Fields (TTS), radiotherapy and chemotherapy with Temozolomide (TMZ), which results in a median survival of only 20 months^1^. This therapeutic resistance is mainly attributed to the inherently invasive nature of GBM cells, which enables their infiltration into the surrounding brain parenchyma, and the pronounced heterogeneity of the tumor microenvironment (TME). Resident microglia and monocytes from the peripheral blood stream constitute a substantial portion, approximately 30%, of the GBM tumor mass^2,3^. Preclinical studies have demonstrated that microglia contribute to a pro-tumorigenic milieu, notably through the induction of platelet-derived growth factor receptor (PDGFR) expression, which subsequently stimulates glioma cell migration and consequently, accelerates tumor progression^4^.

The significant microglial infiltration observed within the glioblastoma microenvironment can be attributed to the secretion of chemoattractant factors by glioma cells. These factors, including colony-stimulating factor 1 (CSF-1), C-X-C motif chemokine 12 (CXCL12 / SDF-1), and granulocyte-macrophage colony-stimulating factor (GM-CSF), stimulate the chemotactic migration of resident microglia towards the tumor mass^5,6^. Furthermore, glioma cells evade microglial phagocytic clearance through the overexpression of the anti-phagocytic “don’t eat me” marker, CD47, effectively inhibiting microglial phagocytic activity. This evasion mechanism contributes to tumor persistence and growth. Additionally, glioma-associated microglia contribute to tumor invasion by secreting matrix metalloproteinase-9 (MMP-9), which facilitates degradation of the extracellular matrix (ECM), thereby promoting glioma cell migration and dissemination to the surrounding brain tissue^7^.

In rat glioma models, microglia has been observed to express arginase-1 (ARG-1) and to be located in the peripheral areas of the induced tumor. This ARG-1 expression within the microglial population has been implicated in the stimulation of tumor cell proliferation^8^. Further, live imaging of rat glioma tissue explants has demonstrated a glioma-induced increase for microglial motility^9^. Additionally, transcriptome analysis of GBMs has shown upregulation of genes that are classically involved in the invasion capability and immunosuppression^10^.

In vitro 2D co-culture models of microglia and GBM cells have demonstrated the critical role of cytokines in GBM progression. Specifically, these co-cultures have revealed that microglia, in the presence of glioma cells, secrete elevated levels of interleukin-6 (IL-6), which subsequently promotes glioma cell migration and invasiveness through the upregulation of matrix metalloproteinase 14 (MMP-14)^11^. While 2D co-culture systems have provided valuable insights into cell-cell interactions, they lack the complex cell-matrix and cell-cell interactions achievable in three-dimensional (3D) co-culture models, which more accurately recapitulate the in vivo TME^12^. Thus, the field has been progressing towards more complex spheroid-based cellular models.

Sørensen et al. investigated the impact of microglia on the sensitivity of GBM homotypic spheroids to TMZ using a transwell co-culture system. Their findings revealed that the presence of microglia conferred increased drug resistance to the GBM spheroids, as evidenced by reduced cell death in response to TMZ treatment^13^. Heinrich et al. increased the level of complexity in their models by developing a tri-culture spheroid model composed of astrocytes, microglia, and glioma cells. Tri-culture spheroids showed an increased proliferation rate as compared to homotypic spheroids. Their study also revealed that astrocytes were the primary cell type contributing to the observed migration^14^. Single-cell RNA-Seq of human tissue sections revealed that human microglia often form a protective pro-tumorigenic niche on the periphery of tumoroids. This contrasts TAMs, which exhibit more infiltrative behavior within the tumoroid core^15^. Thus, this characteristic spatial arrangement might be crucial to recapitulate the native GBM behavior in-vitro.

Here, we developed a humanized heterotypic glioblastoma: microglia 3D spheroid model, using a commercially available tumor-derived glioma cell line (DKMG) or patient derived glioma stem cell line (GB22-13) together with a human-derived microglia cell line (HMC3) which represents 30% of the total cell number. This model served to investigate the impact of microglia on tumor progression, invasion, drug resistance and cellular spatial organization. Our model displayed key characteristics of native GBM and revealing the secretion of specific factors, cytokines, and markers associated with migration, invasion, metastasis, tumor malignancy, and drug resistance, which are absent in homotypic GBM or microglia-only models, underscoring the critical role of co-culturing these cell types.

## Materials and methods

### GSC line generation

A human glioblastoma (grade 4, IDH-1 wild-type, ATRX mut, p53 mut, TERT promoter mut, EGFR amplication and MGMT promoter no metilated) tumor biopsy was obtained from the Neurosurgery Department of Donostia University Hospital (HUD). The project received approval from the Euskadi Ethical Board Committee and the patient provided written informed consent before sample collection. All methods were performed in accordance with the relevant guidelines and regulations. For the generation of the glioblastoma stem cell (GSC) line (GB22-13), the fresh tumor biopsy was cut into small pieces, disaggregated into single cells by placing in accutase (Fisher Scientific) for 15–20 min at 37 °C and filtered with a 70 μm cell strainer^25^. Cells were concentrated by centrifugation and then seeded in complete medium (DMEM / F-12, GlutaMAX™ (Fisher Scientific) supplemented with 20µL / mL of B27 (Fisher Scientific), 10 µL / mL of N2 (Fisher Scientific), D-(+)-Glucose (Sigma Aldrich), P / S (Gibco), 20 ng/mL of Endothelial Growth Factor (EGF, Sigma Aldrich), 20 ng / mL of Fibroblast Growth Factor (FGF, Fisher Scientific) and 10ng / mL of laminin (Sigma Aldrich). Cultures were maintained at 37 °C, 5% CO2 and medium changed every week. The molecular subtype was classified as mesenchymal based on the expression of genes determined by Wang et al.^25^ data not shown).

### Cell culture

DK-MG (AC277) cells were purchased from the German collection of microorganisms and cell cultures GmbH (Leibniz-Institute DSMZ, Braunschweig, Germany). DK-MG cells were seeded at density of 1·10^6^ cell / cm^2^ and cultured in Roswell Park Memorial Institute Medium (RPMI 1640, Gibco, Fisher Scientific) supplemented with 10% heat-inactivated fetal bovine serum (FBS, Gibco, Fisher Scientific) at 37 °C and 5% CO_2_ and cultured until 80% confluence with media changes every second day. Experiments were performed at passage 4.

GB22-13 cells were seeded at 1·10^6^ cell / cm^2^ and cultured in Dulbecco’s Modified Eagle Medium DMEM / F12 Glutamax (Gibco) supplemented with B-27 (20µL / mL), N2 (10 µL / mL), 1% penicillin-streptomycin, 10 ng / mL laminin, 20 ng / mL EGF (ThermoFisher), and 20 ng / mL FGF (PeproTech). Upon 90% confluence, cells were passaged with accutase (Gibco, Fisher Scientifc) at 37 °C, and centrifuged for 4 min at 1200 rpm. After removal of the supernatant, cells were resuspended in complete neurobasal medium containing laminin (20 ng / mL), and media was refreshed every second day.

The human microglia clone − 3 (HCM3) cell line was purchased from the American Type Culture Collection (ATCC) (CRL-3304, VA, USA). HCM3 cells were seeded at a density of 40,000 cell / cm^2^ and cultured in Alpha-Minimum Essential Medium without nucleosides (αMEM, Gibco) supplemented with 10% (v/v) FBS at 37 °C and 5% CO_2_ until 80% confluence. The cell media was replaced every 2 days. Phosphate buffer saline (PBS) was used to wash adherent cells before their detachment with 0.25% Trypsin/EDTA solution (Gibco™).

Human monocytes THP-1 (ATCC) were obtained at passage 0. The cells grew in suspension and were maintained in growth medium composed of RPMI 1640, 2 mM L-glutamine, 1.5 g / L sodium bicarbonate, 4.5 g / L glucose, 10 mM HEPES, 1.0mM sodium pyruvate, 0.05mM 2-mercaptoethanol and 10% FBS. Upon reaching 80% confluency, cells were passaged by centrifugation at 2.5 g for over 5 min, after which the supernatant was removed and the cells resuspended and seeded. Experiments were performed at passage 3.

### Llentiviral production and transduction

Lentiviral production was performed by transfecting HEK293T cells with lentiviral plasmid pMD2.G and pSPAX2 (kindly provided by Didier Trono; Addgene plasmids #12259 and #12260 respectively) and the transfer plasmid tGFP (a gift from Linzhao Cheng; Addgene plasmid #26864). Five hours after transfection, the medium was changed, and viral particles were collected 48 h later and concentrated using Lenti-X Concentrator (Takara Bio). Cells were seeded at 5·10^5^ / well density in 6-well plates and transfected, 48 h later, with 10 µl / well of Lv-tGFP and 2 µl / well of polybrene transfection agent (Merck) for 24 h. Cells, were then selected by the addition of 5 µg / mL puromycin for 4 days, after which a transduction efficacy of > 98% was observed by fluorescence microscopy (Figure S1, supplementary information). Experiments were performed at passage 17.

### Spheroid formation

Ultra-low attachment 96-well plates (Fisher Scientific) were used to generate homotypic and heterotypic spheroids. Spheroids were formed from 30,000 cells for both heterotypic and homotypic spheroids. 30,000 cells were seeded in each well and with a 70:30 GBM: HMC3 cell ratio when heterotypic spheroids were to be formed. Media for heterotypic spheroids was selected after validation in different ratios of DMEM / F12:αMEM media (glioblastoma culture media and microglia culture media) were tested (Supplementary information Figure S2). Spheroids were spontaneously formed after 24 h of culture and maintained in 90 µL DMEM / F12 media supplemented with B-27 (20 µL / mL media ), N2 (21 µL / mL media 1% penicillin-streptomycin, 20 ng / mL, Epidermal Growth Factor EGF (ThermoFisher), and 20 ng / mL fibroblast growth factor hFGF (PeproTech), with a partial media replacement of 50 µL every 2 days.

### Measurements of spheroid morphology from microscopy images

Spheroid size variation and morphological parameters were quantitatively assessed using Image J software. Images were converted to binary format to clearly delineate the spheroid borders. Subsequently, the tool dedicated to particle analysis was used with a specific threshold set for each individual spheroid image to ensure accurate measurements. For each analysis, area, center of mass, perimeter, aspect ratio, diameter and Feret’s diameter were calculated (*n* = 5).

### DNA quantification

Total DNA was quantified for cell proliferation and drug resistance measurements. For proliferation measurements, spheroid samples were prepared and harvested at days 2,5,6 and 7, media was removed and spheroids washed three times with DPBS. For drug resistance analysis quintuplicate samples of homotypic and heterotypic spheroids were formed as described earlier, and harvested after days 2, 5, 6 and 7 of culture. On days 6 and 7, TMZ (MedChemExpress) was added at final concentrations of 0, 200, or 400 µM to the culture media. After 24 or 48 h of exposure, the supernatant was removed, and spheroids were washed three times with DPBS. All samples were kept dry at -80 °C until further use. All samples for DNA quantification were freeze–thawed 3 times and digested by incubation in a 1 mg / mL Proteinase K (Fisher BioReagents™) in Tris/EDTA buffer at 56 °C overnight under orbital shaking. The total DNA content was measured with a CyQuant™ cell proliferation assay kit (Invitrogen), following manufacturer’s instructions and assuming 6.6 × 10^− 6^ µg of DNA per cell to estimate cell numbers. Fluorescence intensity was measured at emission wavelength of 520 nm and excitation wavelength of 480 nm using a BioTek Synergy Neo2 Hybrid Multi-Mode Reader (Agilent).

### EdU staining

To determine which cells were in a proliferative state, Click-iT™ EdU Alexa Fluor™ 647 (Fisher Scientific) was used. Spheroids were formed and stained after 7 days of culture (Supporting Information Figure S3). The staining was initiated the day before the samples were harvested by replacing half of the culture medium with EdU solution to reach a final concentration of 10 µM. Spheroids were incubated overnight at 37 °C inside the incubator. The following day, samples were fixed with 4% PFA for 15 min, washed with a solution of 3% BSA in PBS, and permeabilized with 0,5% Triton-x100 solution for 20 min. Afterwards, 500 µL / well of EdU Click-IT cocktail mix were added and incubated for 30 min protected from the light. Samples were then washed twice with 3% BSA in PBS, and counterstained with Hoechst 33,342 (1:500, 33342) for 25 min, following manufacturer’s instructions.

### Cell staining for cellular Spatial organization

DKMG cells were stained using the Qtracker™ 655 Cell Labeling Kit (Invitrogen). Monolayer cultures of DKMG (1.33 × 10 ^4^ cells / cm^2^) were incubated with the 655-nanocrystal solution (10 nM) at 37 °C for 45 min and 5% CO_2_. Following incubation, cells were trypsinized and immediately used for spheroid formation. GFP positive GB22-13 cells were fixed in 4% paraformaldehyde for 25 min and rinsed three times with DPBS. Samples were then incubated for 30 min with Hoechst 33,342 (1:500) to label DNA and washed three times with DPBS.

### Cell viability

The cell viability was evaluated by LIVE / DEAD™ viability kit for mammalian cells (Invitrogen), following manufacturer’s instructions. Briefly, culture media was removed and the spheroids were washed 2 times with DPBS. Afterwards, 1 mL of 6 µM ethidium homodimer and 1 µl of 1 mM Calcein were combined and 80 µL of the solution was used to cover each spheroid. The cells were incubated for 30 min protected from light and the staining solution was replaced with DPBS. The samples were visualized within 45 min after staining.

### Fluorescence and optical microscopy

Samples for LIVE / DEAD assay, cell invasion, cellular organization of DKMG and THP-1 polarization were recorded using an Eclipse Ti2 Microscope (Nikon) equipped with a LED-based Lumencor Spectra II Illuminator and a large field Photometrics Iris 15 sCMOS camera in epifluorescence mode. Samples for EdU staining and cellular organization using GFP-GB22-13 were visualized using a Light Scanning Microscope 880 Airyscan (Zeiss) equipped with Ar, DPSS and HeNE lasers, and PMT photomultiplier and GaAsP detector using µ-Slide 15 Well 3D Glass Bottom (Inycom Biotech). Optical microscopy images were recorded on an inverted Nikon Eclipse Ts2 microscope with phase contrast.

### Monocyte polarization and immunostaining

THP-1 human monocytes were cultured with spheroid conditioned media collected 24 h after refreshing and after 7 days of spheroid culture in 24-well adherent plates. THP-1 cells were seeded in a 1:1 mixture of RPMI 1640 and spheroid media at a density of 700,000 cell / cm2. After 24 h, cell adhesion was evaluated by fluorescence microscopy. Control samples to evaluate the staining of THP-1-derived macrophages towards CD163 and CD68 expressing cells were also prepared. To differentiate THP-1 monocytes towards macrophages a protocol previously established in the literature was used^75^. In brief, THP-1 monocytes were seeded on p24 well-plates at a density of 3 × 10^5^ cells/well. Monocytes were stimulated with 10 ng/mL phorbol 12-myristate 13-acetate (PMA, Thermo Scientific Chemicals) dissolved in RPMI 1640 medium. Cells were incubated with PMA for 24 h at 37 °C in a humidified atmosphere containing 5% CO_2_. Afterwards, adhered cells were polarized towards pro-inflammatory M1 phenotype by incubation with 20 ul/ml media of lipopolysaccharide (LPS) 50ng/ml for 48 h. Anti-inflammatory M2 polarization was induced by incubation with 20 ng/ml of human interleukin 4 (IL-4) (Prepotech) and 20 ng/ml of human interleukin 13 (IL-13) (Prepotech) for 72 h. To that end, samples and controls were washed twice with DPBS and the cells were fixed by incubation in a 4% paraformaldehyde (PFA) solution in PBS for 15 min. The cells were then permeabilized in a solution of 0.1% Triton X-100 (Fisher BioReagents) in PBS for 15 min and then washed again twice with PBS. Samples were then blocked for 1 h at RT in a solution of 3% bovine serum albumin (BSA) and 0.01% Triton X-100 in PBS. After rinsing with PBS, samples were incubated overnight at 4 °C with primary antibodies: rabbit-anti-cd86-Alexa Fluor 647 (1:50, Abcam ab288358) and mouse-anti-cd163 (1:100, Abcam ab156769). Samples were then rinsed with a solution of 0.3% BSA and 0.001% Triton X-100 in PBS and incubated for 1 h at RT with Alexa Fluor–conjugated secondary antibodies (1:200). Afterwards, samples were stained with Phalloidin Alexa Fluor 568 (Invitrogen, 1:100) for 2 h, washed with PBS and incubated with Hoechst 33,342 (1:500) for 15 min and lastly washed with PBS. The samples were kept in PBS at 4 °C until imaging by confocal microscopy.

### GelMA synthesis and characterization

GelMA was synthesized using porcine skin gelatin (Merck, Type A, 300 Bloom) according to Loessner et al.^17^ (Supplementary Information Figure S4, A). In brief, a 10% (w/v) gelatin solution was prepared by dissolving 3 g of gelatin in 27 mL of distilled water at 37 °C, under continuous stirring for 1 hour. The pH of the solution was adjusted to 9 using 2 M NaOH. Subsequently, methacrylic anhydride (MAA, Merk) was added dropwise to the gelatin solution at a ratio of 0.6 mL / g. The reaction proceeded for 3 h at 50 °C, with continuous magnetic stirring and protected from light. The pH was maintained at 9 throughout the reaction by the addition of 2 M NaOH. The resulting solution was centrifuged at 5 g for 5 min, and the supernatant was collected. The supernatant was then diluted three-fold with distilled water and dialyzed against distilled water for 5 days at RT using SnakeSkin dialysis membranes (10 kDa MWCO, 35 mm diameter, Fisher Scientific). Dialysis water was changed daily. The dialyzed solution was sterile-filtered through a 0.22 μm membrane filter and subsequently lyophilized for 5 days under sterile conditions. The lyophilized GelMA was stored at -40 °C. Proton nuclear magnetic resonance (H^1^-NMR) was used to validate the methacrylation (Supporting Information Figure S4, B). Samples were prepared by dissolving 5 mg of the liophylized powder in 700 µl of D_2_O (Fisher Scientific Chemicals). NMR spectra were recorded on a 500 MHz Bruker advance spectrometer (Bruken bioSpin) applying a water suspension pulse sequence.

### Spheroid migration on GelMA

After 5 days of formation, GBM spheroids were transferred to a 4% (w/v) GelMA hydrogel layer formed on a 96 well plate that previously photo-crosslinked with ultra-violet light for 4 min. Spheroid images were acquired using optical microscopy on days 5, 6, and 7 and cell invasion was assessed by measuring the increase in the radius of the projected area of cells covering the surface of GelMa as compared to the radius of the original spheroid projected area. Radial invasion was quantified using ImageJ, and the invasion distance was expressed as an average fold change in radius as compared to day 0.

### Flow cytometry

20 spheroids of each condition were prepared as detailed above, harvested after 5,6 and 7 days with low-retention tips and transferred to microcentrifugation tubes. Samples were then centrifuged at 1200 rpm for 5 min. After centrifugation (Centrifuge 5702, Eppendorf), the supernatant was carefully removed and 500 µL Accutase (Gibco) were added to the spheroids and mixed by pipetting up-down. The spheroids were then incubated for 30 min at 37 °C in an orbital shaker for dissociation. Samples were mixed by pipetting up-down again to dissociate potentially left aggregates and the suspensions were centrifuged at 1200 rpm for 10 min. The cell suspensions were then fixed with 4% (v/v) PFA solution in PBS for 20 min. Fixed cells were centrifuged and stained with Hoechst 33,342 (1:500) for 15 min and lastly washed with PBS. The samples were kept in PBS at 4 °C until quantification. Flow cytometry was conducted using a Beckman Coulter CytoFLEX flow cytometer equipped using a 405 nm laser and a 450/45 bandpass filter to detect Hoescht and a 488 nm laser and a 525/40 bandpass filter for GFP detection.

### THP-1 and THP-1-derived macrophage migration in response to spheroid secretome

Three heterotypic or homotypic GB22-13-based spheroids were cultured on the bottom of a 24-well plate after 5 days of formation. A transwell insert with an 8 μm pore diameter membrane (Corning) was placed on the wells and 1 × 10^5^ THP-1 monocytes or 1 × 10^5^ THP-1-derived macrophages were added on top. Complete neurobasal media was added at the bottom of the transwell and complete THP-1 media on the top. After 48 h of culture, the amount of migrated cells was assessed by staining with crystal violet, following manufacturer instructions. Stained transwells were visualized using an optical inverted microscope.

To differentiate THP-1 monocytes towards macrophages a protocol previously established on the literature was used^75^. In brief, THP-1 monocytes were seeded on p24 well-plates at a density of 3 × 10^5^ cells/well. Monocytes were stimulated with 10 ng/mL phorbol 12-myristate 13-acetate (PMA, Thermo Scientific Chemicals) dissolved in RPMI 1640 medium. Cells were incubated with PMA for 24 h at 37 °C in a humidified atmosphere containing 5% CO_2_.

### Proteome profiler cytokine array

Spheroids supernatants (GBM, co-culture, microglia) were collected after 7 days of culture, and stored at -80 °C until further use. The conditioned supernatants were assayed with Proteome Profiler Human XL cytokine Array Kit (R&D systems) according to the manufacturer’s protocol. The three microarrays were exposed for 5 min to X-ray radiation and imaged using an IBright 1500 system (Invitrogen). Mean gray values of technical duplicates were measured using ImageJ Software.

### Statistical analysis

All the data was statistically analyzed using GraphPad Prism 8 Software. Biological experiments used at least five biological replica, including microscopy images. One-way and two-way analyses of variance (ANOVA) were carried out with Tukey’s multiple comparisons test; (****) *p* < 0.0001, (***) *p* < 0.001, (**) *p* < 0.01, and (*) *p* < 0.1 was used All the results were expressed as mean ± standard deviation.

## Results

### Establishment of a 3D Microglia-Glioma Co-culture model

**Fig. 1:**
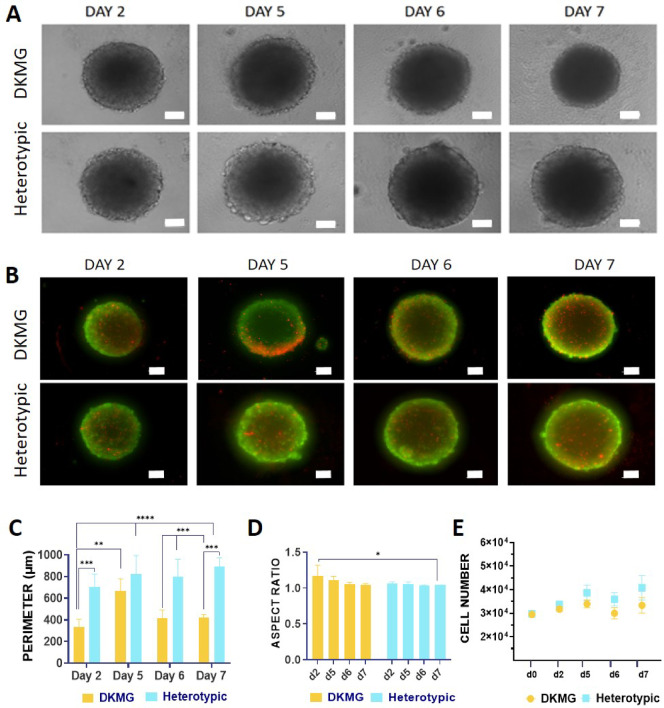
Establishment and characterization of DKMG homotypic (glioma) and heterotypic (glioma: microglia) spheroids. (**A**) Optical microscopy images of DKMG homotypic and heterotypic spheroids at days 2, 5, 6 and 7 of culture. (**B**) Fluorescence microscopy images of DKMG homotypic and heterotypic spheroids at days 2, 5, 6 and 7 of culture stained for calcein (live, green) and ethidium homodimer-1 (dead, red). Perimeter length (**C**) and aspect ratio (**D**) of DKMG-based spheroids over 7 days of culture. (**E**) Cell number as calculated from total DNA quantification of DKMG-based spheroids over 7 days of culture. Error bars show average ± standard deviation. Statistical significance was calculated from *n* = 5 spheroids with a two-way ANOVA with posthoc Tukey’s multiple comparison tests; (****) *p* < 0.0001, (***) *p* < 0.001, (**) *p* < 0.01, and (*) *p* < 0.1.

**Fig. 2:**
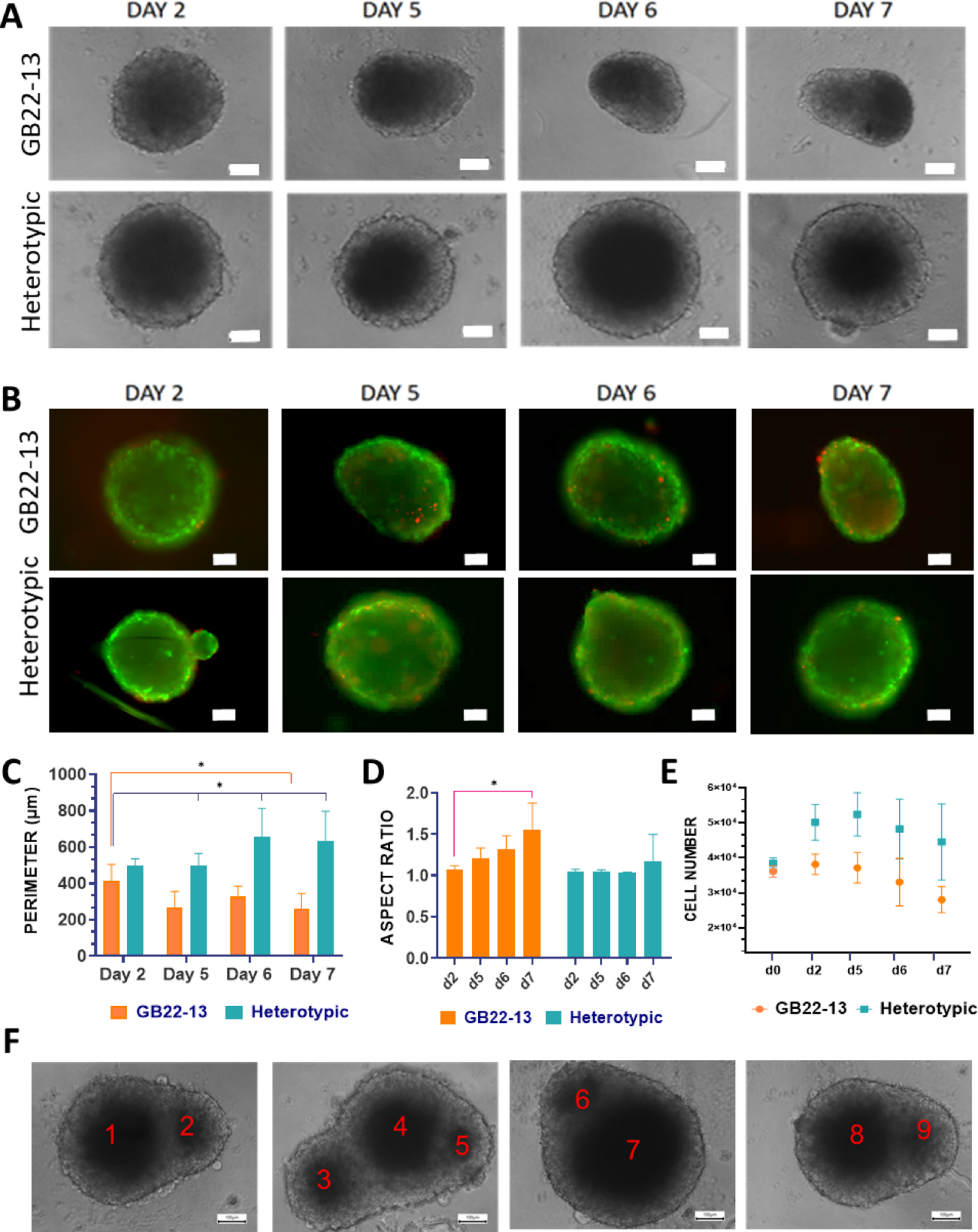
Establishment and characterization of GB22-13 homotypic (glioma) and heterotypic (glioma: microglia) spheroids. (**A**) Optical microscopy images of GB22-13-based homotypic and heterotypic spheroids at days 2, 5, 6 and 7 of culture. (**B**) Fluorescence microscopy images of GB22-13-based homotypic and heterotypic spheroids at days 2, 5, 6 and 7 of culture stained for calcein (live, green) and ethidium homodimer-1 (dead, red). Perimeter length (**C**) and aspect ratio (**D**) of GB22-13-based spheroids over 7 days of culture. (**E**) Cell number as calculated from DNA quantification of GB22-13-based spheroids over 7 days of culture. (**F**) Optical microscopy images of multinucleated heterotypic spheroids. Scale bar is 100 μm for all images. Error bars show average ± standard deviation. Statistical significances of spheroids (*n* = 5) measurements were calculated from two-way ANOVA with posthoc Tukey’s multiple comparison tests; (****) *p* < 0.0001, (***) *p* < 0.001, (**) *p* < 0.01, and (*) *p* < 0.1 was used.

The role of microglia in GBM progression, malignancy, immune evasion and drug resistance has been established in-vivo and validated in-vitro^13,18^. However, in-vitro models are scarce, vaguely characterized in terms of their capability to replicate key features of GBM, and generally make use of cell lines such as U87 that, oftentimes, display growth characteristics that deviate from patient derived glioma cells^14,19^. Therefore, 3D GBM models were generated using both a commercially available human cell line, DKMG, as well as a primary glioma stem cell line (GSCs) isolated from a GBM patient after tumor resection, GB22-13. Glioma cells were combined with the human microglia cell line HMC3 to form heterotypic spheroids in a 70:30 glioma: microglia cell ratio, following in-vivo evidence that demonstrates a 70:30 cell ratio in native GBM tumors, augmenting the relevance of our model^10,20^. Spheroids were generated using ultra-low attachment plates, which promoted spontaneous cell aggregation. After two days, the formation of GBM spheroids was confirmed in all conditions; homotypic DKMG or GB22-13 spheroids and heterotypic DKMG: HMC3 and GB22-13:HMC3 spheroids (Fig. 1A- and 2 A). Cells were viable over culture periods of 7 days with no evident formation of a necrotic core (Figs. 1B and 2B). DKMG-based spheroids, both homotypic and heterotypic, presented a higher amount of dead cells that were distributed across the entire volume as compared to spheroids based on GB22-13 cells.

Heterotypic DKMG-based spheroids, showed an increased perimeter with rougher edges of loosely attached proliferating cells over the entire culture period, reaching values of 891 ± 82,4 μm after 7 days, as compared to a perimeter of 425 ± 26,3 μm in homotypic spheroids at the same culture period (Fig. 1C). The aspect ratio of the spheroids remain constant at values near 1, indicative of an spherical shape (Fig. 1D).

Cell number in DKMG spheroids increased over the first 5 day of culture in homotypic spheroids, reaching a plateu, with values of 3.3·10^4^ ± 3.3·10^3^ cells after 7 days of culture. Heterotypic spheroids, however, kept proliferating until day 7 with 40,8·10^4^± 5.2·10^3^ cells per spheroid and a resulting 1.4 ± 0.3 fold increase in cell number as compared to DKMG cells alone (Fig. 1E). Despite the increased cell number, no significant increase in diameter was detected for either spheroid type, suggesting a densification, which is supported by the appearance of a darker nuclei in optical microscopy images (Supporting Information Figure S2, A and Figure S5) Nevertheless, no significant increase in solidity was detected on either spheroid type (Supplementary Information Figure S6). Spatial analysis of proliferating cells by EdU staining revealed a greater number of proliferative (*EdU*^*+*^) cells on the periphery of the spheroid (Figure S3), although the core of the spheroids also demonstrated a significant number of proliferating cells, without a clear formation of a necrotic core.

GB22-13-based spheroids demonstrated a behavior that was comparable to DKMG-based spheroids. Heterotypic spheroids exhibited larger perimeters that increased over time with values of 634 ± 163 μm after 7 days of culture as compared to 261 ± 83 μm for homotypic spheroids, suggesting again a densification of the cell aggregate (Fig. 2C). In contrast to DKMG-based spheroids, GB22-13 homotypic spheroids tended to acquire a peanut-like shape (Fig. 2A and B) with aspects ratios peaking 1.5 after 7 days of culture (Fig. 2D).

Cells proliferated only the first 5 days in both homotypic and heterotypic spheroids, when a plateau was reached. Yet, the cell number was higher for heterotypic spheroids, with 4.4.·10^4^ ± 10·10^3^cells per spheroid after 7 days of culture as compared to 2.8·10^4^ ± 3.9·10^3^ cells for homotypic ones (Fig. 2E). Surprisingly, despite the overall lower aspect ratio of around 1 and the invariable cell diameter (Supplementary Information Figure S5), heterotypic spheroids developed multinucleated structures with two or more densification areas (darker centers observed by optical microscopy) coexisting on a single spheroid. Multinucleated structures could be observed already at day 5 of culture, with 1/3 of the spheroids presenting various densification nuclei after 7 days of culture. The formation of multinucleated structures suggests a potential microglial influence on cell fusion or division (Fig. 2F). Thus, the presence of microglia resulted, when combined with either glioma cell type, in an increased cell proliferation and spheroid perimeter.

To decipher the potential contribution of microglia and glioma cells to cell proliferation on heterotypic spheroids, flow cytometry was conducted. Upon culture of heterotypic GB22-13-based spheroids, the initial 90:10 glioma: cell ratio was lost, showing an increased microglia population with cell ratios of 60:40 and 56:44 after 2 days and 5 days of culture, respectively (Supplementary Information Figure S7A-D). Thus, the increased cell proliferation in heterotypic spheroids could primarily be attributed to HMC3 proliferation.

### Microglia enhances the invasiveness of GBM models

**Fig. 3:**
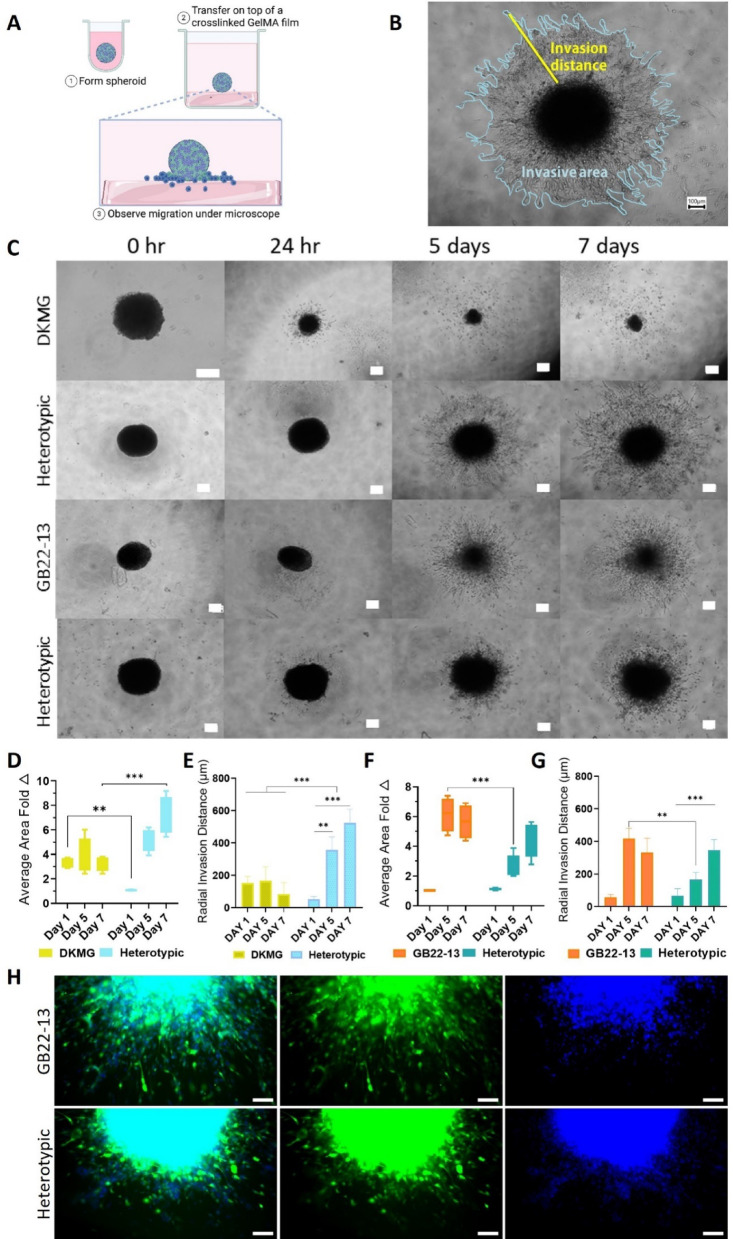
Spheroid invasiveness on GelMA films. (**A**) Schematic representation of the experimental setup for evaluating spheroid invasion. (**B**) Representative image of the process to evaluate the invasive area and invasion distance from optical microscopy images. (**C**) Optical microscopy images of homotypic and heterotypic spheroids based on DKMG and GB22-13 cells let grow on top of GelMA films over 7 days, showing the invasion process. Average area fold change for both homotypic and heterotypic spheroids over 7 days of culture for DKMG (**D**) and GB22-13-based (**F**) spheroids. Radial invasion distance over culture time of homotypic and heterotypic spheroids based on DKMG (**E**) and GB22-13 (**G**) cells. (**H**) Fluorescence microscopy images of homotypic and heterotypic spheroids on top of the GelMA films using GFP-GB22-13 cells (green) and counterstained for DNA (all cells, Hoechst, blue). Scale bar is 100 μm for all images. Error bars show average ± standard deviation. Statistical significance was calculated from *n* = 5 spheroids with a one-way ANOVA with posthoc Tukey’s multiple comparison tests; (****) *p* < 0.0001, (***) *p* < 0.001, (**) *p* < 0.01, and (*) *p* < 0.1.

Another key characteristic of GBM tumors is the strong invasive potential, with cells migrating out of the tumor and proliferating in a new area of the brain where a secondary tumor might be formed. To investigate the impact of microglia on GBM invasiveness, the capability of cells within spheroids to escape and proliferate on an ECM mimicking substrate material was evaluated. To do so, pre-formed spheroids were cultured on top of cross-linked methacrylated gelatin (GelMA) hydrogel films, following a method previously reported (Fig. 3A)^14,21^. To quantify invasion, the invaded area and invasion distance were measured. The invaded area was calculated as the increased projected area occupied by cells as compared to the initial projected area of the spheroid alone at day 0. And, the invasion distance was quantified as the most distant point occupied by a cell from the center of the spheroid at several locations of the perimeter of the invaded area as compared to the initial projected diameter of the spheroid alone at day 0 (Fig. 3B). Homotypic and heterotypic spheroids displayed distinct invasion patterns (Fig. 3C). DKMG homotypic spheroids initiated invasion rapidly, within 24 h, displaying a radius and area of invasion of 153 ± 51 μm and 9.2·10^4^ ± 6.9·10^3^ µm^2^, respectively, that was maintained constant over the 7 days culture period (Fig. 3D and E). In contrast, DKMG-based heterotypic spheroids presented an initial slower invasion, with a radius and area of invasion of 51 ± 13 μm and 1.7·10^5^± 3.3·10^4^ µm^2^, respectively, that accelerated over time, reaching values of 497 ± 144 μm radius and 1.14·10^6^± 72.4·10^3^ µm^2^ area of invasion, respectively, after 7 days of culture. Thus, the presence of microglia in DKMG heterotypic spheroids resulted in a 7,1 ± 1,5-fold increased area of invasion as compared to homotypic DKMG spheroids.

The effect of microglia in GB22-13-based spheroids appeared to be less pronounced as in DKMG-based spheroids but presented a similar trend (Fig. 3C). The radius and area of invasion for both homotypic and heterotypic spheroids after 24 h was lower than that observed for DKMG-based spheroids, with values of 63 ± 14 μm and 9.2·10^4^± 6.9·10^3^ µm^2^, and 76 ± 28 μm and 1.5·10^5^± 2.3·10^4^ µm^2^, respectively (Fig. 3F and G). After 5 days of culture, the invasion was evident for both, homotypic and heterotypic spheroids, but higher for the former. Heterotypic spheroids presented a radius and area of invasion of 172 ± 42 μm and 3.3·10^5^ ± 9.5·10^4^ µm^2^, while a radius and area of invasion of 431 ± 66 μm and 5.9·10^5^ ± 1.1·10^5^ µm^2^ were measured for the homotypic spheroids. However, the radius and area of invasion seemed to reach a plateau after 7 days for homotypic spheroids, while microglia-containing spheroids continued invading the ECM-like matrix. After 7 days of culture, homotypic and heterotypic spheroids presented a radius and area of invasion that were not significantly different amongst them, with values of 399 ± 109 μm and 5.2·10^5^ ± 1.1·10^4^ µm^2^, and, 334 ± 48 μm and 5.3·10^5^ ± 9.2·10^4^ µm^2^, respectively.

HMC3 cells and patient-derived GB22-13 cells present different proliferation rates when cultured in monolayer, with HMC3 being significantly faster. Thus, to be able to decipher what type of cell, microglia or glioma, was responsible for the invasion in heterotypic spheroids, GFP-labelled GB22-13 cells (GFP-GB22-13) were used to form heterotypic spheroids (Fig. 3H and Supporting Information Figure S8). After 7 days of culture on GelMa substrates, the invasion was mainly attributable to microglia cells (labelled only for DNA, blue) with few glioma cells present outside the tumoral body. This suggests that in the presence of microglia, glioma cells decrease their proliferation rate or are simply confined in the core of the tumor as evidenced by others^22^.

### Heteroypic spheroids display an increased drug resistance and clonogenicity after treatment

Previous research highlights the critical role of microglia in modulating glioma cell behavior and drug sensitivity. Notably, microglia has been shown to induce an interferon-stimulated gene expression profile in GBM cells, a factor that could significantly influence the efficacy of TMZ^13^. Furthermore, studies indicate that microglia contribute to TMZ resistance by activating STAT3 through the release of IL-11. This signaling pathway promotes an M2 polarization of microglia, consequently generating an immunosuppressive tumor microenvironment (TME) that can hinder therapeutic outcomes^23^. Thus, the potential of homotypic and heterotypic spheroids to survive after treatment with TMZ, the gold-standard chemotherapeutic, was evaluated in a comparative manner. To the best of our knowledge, the drug sensitivity of DKMG and patient-derived glioma cells has not been reported yet. Therefore, based on a comprehensive review of existing literature, TMZ drug concentrations ranging from 0 µM to 400 µM and exposure times of 24 and 48 h were tested. This range is in concordance with the wide variability of TMZ sensitivity observed across different GBM cell lines. Specifically, it has been previously reported that glioma cell lines exhibit sensitivity to a broad spectrum of TMZ concentrations, with an average effective exposure ranging from 120 to 220 µM and some reaching median IC50 values as high as 800 µM after 72 h of treatment^19^.

**Fig. 4:**
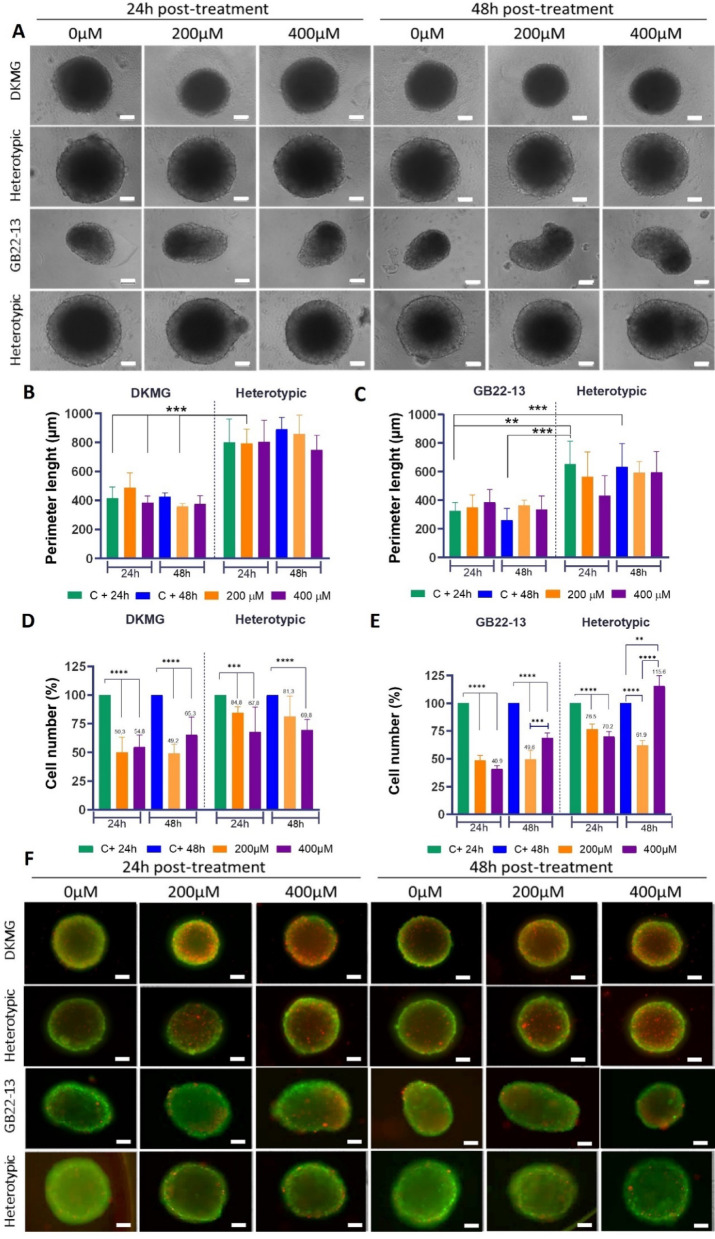
Spheroid response to TMZ exposure. (**A**) Optical microscopy images show morphological differences observed in both DKMG or GB22-13 homotypic and heterotypic spheroid models following 24 and 48 h of exposure to 200 and 400 µM TMZ. Perimeter of DKMG-based (**B**) and GB22-13-based (**C**) spheroids after TMZ exposure. (**D** and **E**) Cell viability after TMZ treatments in both homotypic and heterotypic spheroids of DKMG (**D**) and GB22-13 (**E**). (**F**) Live / Dead fluorescence microscopy images of spheroids after drug exposure (green, alive and red, dead). Scale bar is 100 μm. Error bars show average ± standard deviation. Statistical significance was calculated from *n* = 5 spheroids with a two-way ANOVA with posthoc Tukey’s multiple comparison tests; (****) *p* < 0.0001, (***) *p* < 0.001, (**) *p* < 0.01, and (*) *p* < 0.1.

DKMG homotypic spheroids showed a decreased size after 24 h of exposure to 200 µM TMZ, which was recovered after 48 h. (Fig. 4A), while exposure to 400 µM resulted on no significant variation in the size. Nevertheless, the perimeter of homotypic spheroids remained constant or nor significantly different upon treatment (Fig. 4B). Conversely, DKMG heterotypic spheroids maintained a larger size and a higher perimeter, with no significant decrease in size, nor perimeter regardless of the TMZ dose or exposure time.

Contrary to DKMG-based spheroids, GB22-13 homotypic spheroids retained a rough perimeter and the peanut-like shape observed earlier, with no apparent size reduction upon TMZ treatment (Figs. 1A and 4A). GB22-13 heterotypic spheroids, however, presented a reduced size and perimeter after 400 µM TMZ exposure for 24 h, which was again recovered after 48 h of treatment (Fig. 4A and C). Interestingly, Heterotypic GB22-13 spheroids continued to form multinucleated structures even after drug treatment (Fig. 4A).

Quantification of the total percentage of cells remaining after TMZ treatment showed that homotypic spheroids of both DKMG and GB22-13, presented a consistent decrease in cell number after 24 h of drug exposure (Fig. 4D and E). Specifically, DKMG homotypic spheroids exhibited a cell number that was 50 ± 13% of the non-treated control samples after treatment with 200 µM TMZ and 55 ± 10% for a higher dose of 400 µM and, GB22-13 homotypic spheroids of 49 ± 4% of the non-treated control samples after treatment with 200 µM and 40 ± 3% with 400 µM. In contrast, heterotypic spheroids maintained significantly higher cell viabilities after 24 h. DKMG heterotypic spheroids showed a total cell number that was 84 ± 5% of that of non-treated samples after treatment with 200 µM TMZ and of 68 ± 21% with 400 µM. Similarly, GB22-13 heterotypic spheroids presented a total cell number of 76 ± 4.8% of that of non-treated samples after treatment with 200 µM TMZ and of 70 ± 4.2% with 400 µM. Interestingly, in all spheroid models, total cell number began to increase after 48 h of exposure, with this trend being particularly notable in heterotypic models of GB22-13 spheroids exposed to 400 µM TMZ for 48 h, where the percentage of remaining cells reached 116 ± 9.1%, indicative of a proliferation that was higher than their counterparts exposed to 0 µM TMZ. This unexpected increase directly correlates with our observation of the formation of multinucleated spheroids, even after drug treatment. Nevertheless, measurements of total cell number fail to discriminate among cell types and, thus, whether microglia of glioma cells were preferentially proliferating on these models is not known.

These data was supported by live/dead imaging where a higher amount of dead cells is observed on DKMG homotypic and heterotypic spheroids, while GB22-13 spheroids, present fewer dead cells (Fig. 4F).

### Spatial organization of microglia and its impact on drug penetration

Following the observations of the formation of multinucleated structures (Fig. 4F), lower glioma cell migration during invasion on ECM-like substrates (Fig. 3C) and increased drug resistance (Fig. 4D-E), we hypothesized that spatial cell organization or confinement of glioma cells might be occurring within heterotypic spheroids, as observed earlier in in-vivo scenarios, promoting a selective cell death upon TMZ treatment^22^.

**Fig. 5:**
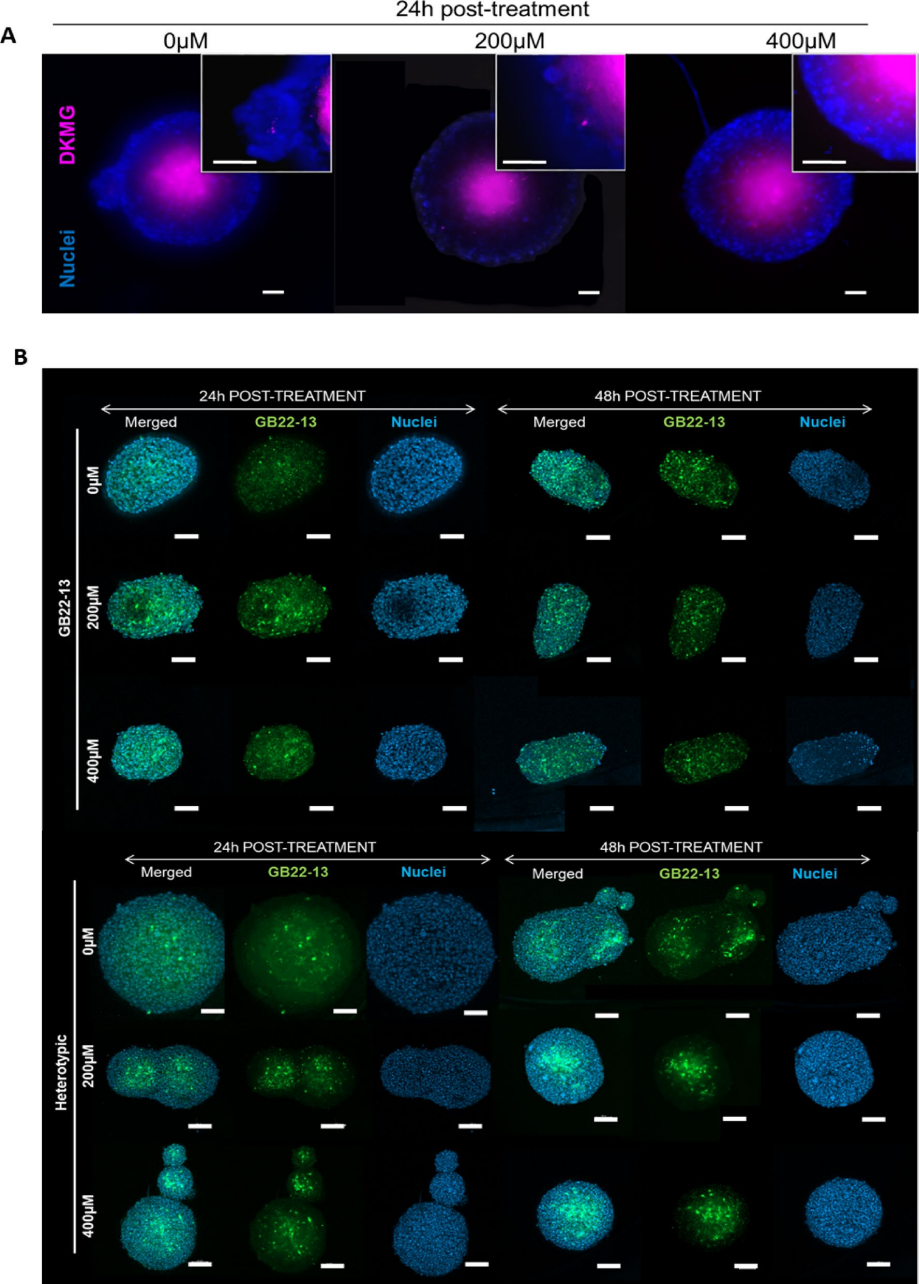
Spatial organization on heterotypic spheroids. (**A**) Fluorescence microscopy images show cellular spatial organization within DKMG heterotypic spheroids after exposure to 0, 200 and 400 µM TMZ for 24 h. DKMG glioma cells are labeled in pink with Qtrack655, and all cells stained for DNA (Hoechst, blue). (**B**) Confocal microscopy images of GFP-GB22-13 heterotypic models after treatment with 0, 200 and 400 µM TMZ for 24 and 48 h. GFP-GB22-13 glioma cells are visualized in green, and all cells are stained for DNA (Hoechst, blue). Scale bar is 100 μm in all images.

To investigate the spatial organization and specific effect of TMZ on either microglia or glioma cells on the heterotypic spheroids, we utilized GFP-GB22-13 cells and DKMG cells fluorescently labeled with a cell tracker to form spheroids. Fluorescence microscopy imaging revealed that, indeed, both DMKG-based and GB22-13-based heterotypic spheroids presented a core-shell cell organization with the core composed of mainly glioma cells and the shell of microglia cells (Fig. 5A). Interestingly, multinucleated GB22-13-based spheroids that formed spontaneously during culture presented also this core-shell organization, with glioma cells in the core surrounded by a shell of microglia cells, even after treatment with TMZ (Fig. 5B). This spatial organization suggests a potential mechanism by which microglia may hinder drug penetration and contribute to the observed TMZ resistance. Nevertheless, a careful observation of the cellular spatial organization during TMZ treatment fails to reveal any predominant decrease in either the microglia shell, nor the tumoral core.

To further investigate the contribution of the presence of microglia on the observed drug resistance and cell proliferation after treatment with TMZ on heterotypic spheroids the variation on the ratio of microglia: glioma cells before and during exposure to TMZ was quantified via flow cytometry (Supplementary Information Figure S7). Our results show that after 24 h of drug exposure, the percentage of GB22-13 viable cells decreased from 37.8% (0 µM) to 22.4% (200µM) and 23.5% (400 µM). This result indicates a 77.6% and 76.5% of microglia cells in the spheroids after treatment with 200 µM and 400 µM TMZ, respectively, as compared to the initial 66.2%. Thus, GB22-13 cells suffer a decrease of 40.7% (from a 37.8% to a 22.4%) and 37.8% (from 37.8% to 23.5%) with respect to the number of glioma cells in non-treated spheroids (0 μm), while the percentage of microglia cells incraeses 24.7% and 22.9% with respect to non-treated spheroids after the same time of culture (0 μm). Thus, the sensitivity of GB22-13 to TMZ appears to be higher than that for HMC3 cells, which would validate the idea of the protective character of the microglia shell observed when putting it into context of the total cell number measured.

### Microglia containing GBM models differentiate monocytes into anti-inflammatory macrophages

A key characteristic of glioma tumors is their capability to escape the immune system. Upon vascularization, peripheral blood monocytes enter the tumoral space where they are polarized towards macrophages presenting anti-inflammatory (M2) phenotypes; interfering with the innate immunity and thus, contributing to immune evasion when tumoral cells are able to proliferate and escape the immune response.

**Fig. 6:**
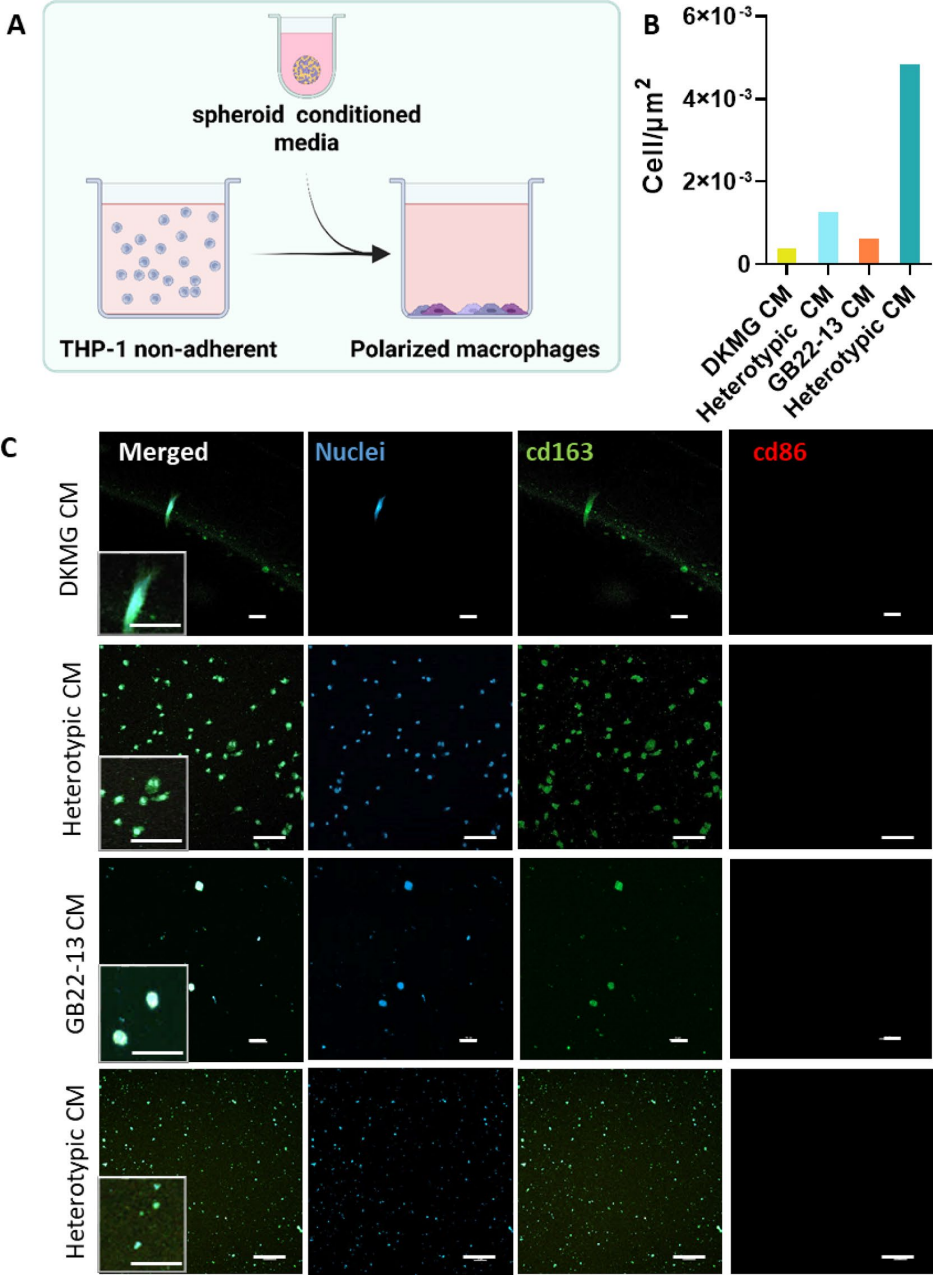
Impact of microglia on the immune evasion capability. (**A**) Schematic representation of the experimental process of monocytes exposed to spheroid (homotypic/heterotypic) conditioned media (CM). (**B**) Number of THP-1-derived macrophages adhered / µm^2^ after exposure to CM as calculated from the images in (C). (**C**) Immunofluorescence images of THP-1 monocytes cultured with CM for 48 h. Cells were stained for CD163 (green, anti-inflammatory), CD86 (red, pro-inflammatory) and DNA (blue). The scale bar is 100 μm in all images.

To investigate the impact of microglia within GBM heterotypic spheroids on the potential of the model to interfere with the innate immunity and contribute to immune evasion, human peripheral blood monocytes (THP-1) were exposed to spheroid-conditioned media (CM) (Fig. 6A). THP-1 cells grow on suspension and attach to the substrate upon differentiation towards macrophages, that can present either anti-inflammatory (M2) or pro-inflammatory (M1) phenotypes (Figure S9). The addition of CM from homotypic spheroids to THP-1 cultures resulted in the differentiation of few cells, as observed from fluorescence microscopy images (Fig. 6B). In contrast, the culture of THP-1 monocytes with CM from heterotypic spheroids, both DKMG-based and GB22-13-based, resulted in a high amount of cells differentiated into macrophages. Interestingly, all visualized macrophages polarized towards an anti-inflammatory phenotype, evidenced by the expression of CD163 and the lack of expression of CD68 (Fig. 6C). These results suggest a more immunosuppressive behavior of GBM when in the presence of microglia through interference with the innate immune system.

### Monocyte cell migration is stimulated by microglia

**Fig. 7:**
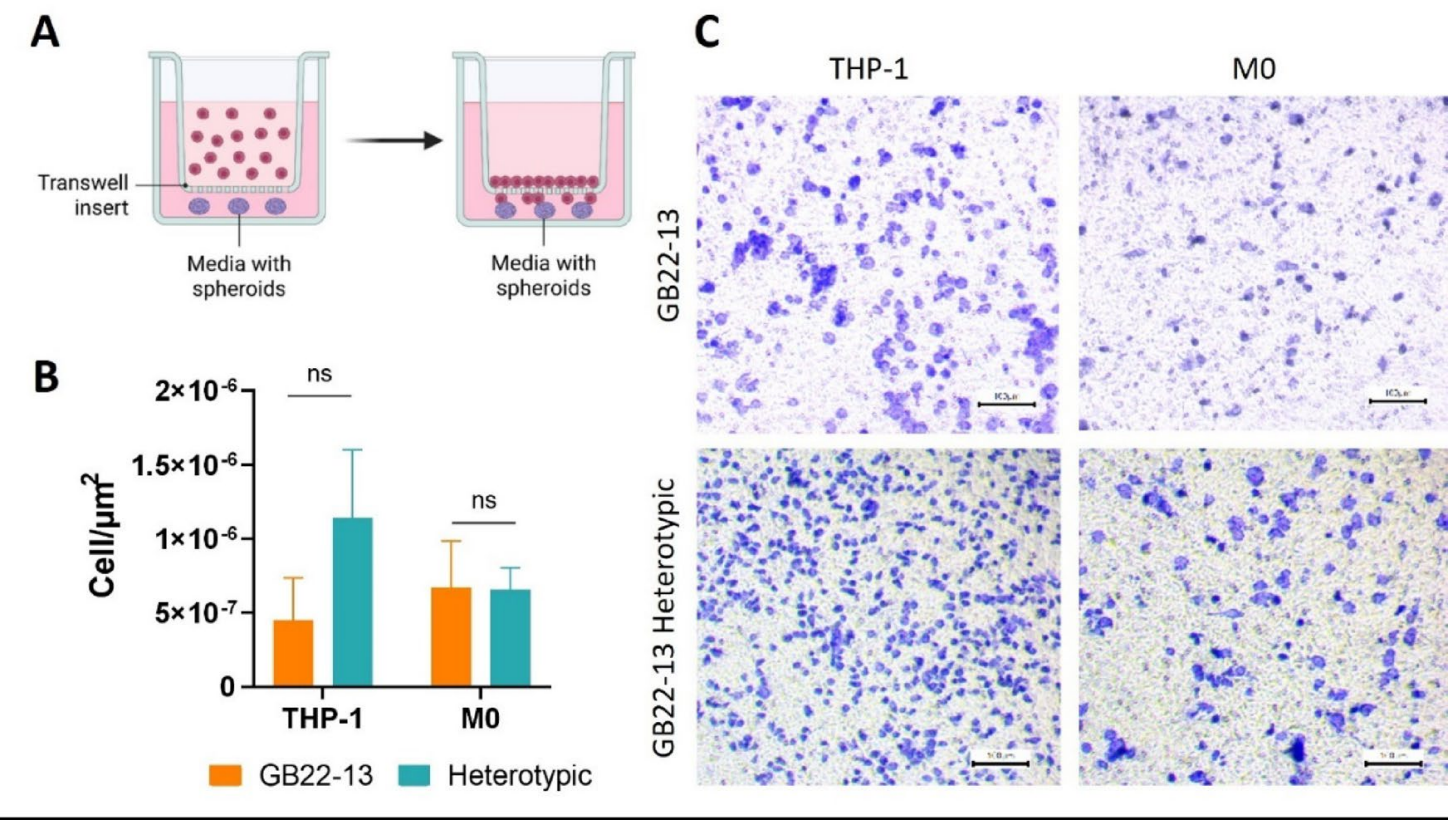
Migratory behavior of monocytes in response to homotypic and heterotypic spheroids. (**A**) Schematic illustration of the transwell experimental setup used for assessing THP-1 migration and polarization in the presence of spheroids (*n* = 2). (**B** Quantification of THP-1 and chemically differentiated macrophages (M0) from transwell inserts. (**C**) Optical microscopy images of THP-1 cells and chemically differentiated THP-1-derived macrophages stained with crystal violet after crossing the transwell insert upon 48 h of co-culture with heterotypic and homotypic spheroids. Scale bar is 100 μm. Statistical significance was calculated from *n* = 3 with a two-way ANOVA with posthoc Tukey’s multiple comparison tests; (****) *p* < 0.0001, (***) *p* < 0.001, (**) *p* < 0.01, and (*) *p* < 0.1.

Next, the potential of heterotypic spheroids to enhance a migratory response in THP-1 human monocytes was investigated. To do so, a transwell system was used to co-culture three heterotypic or homotypic spheroids (bottom part) with either THP-1 monocytes in suspension or THP-1 cells chemically differentiated towards macrophages, on top of the transwell insert (Fig. 7A). Having demonstrated the higher capability of GB22-13 patient derived cells to reflect key characteristics of GBM tumors, such as the formation of multinucleated structures, even after treatment with the chemotherapeutic drug TMZ, the following analysis were performed only with GB22-13 cell-based spheroids.

After 2 days of co-culture, quantification of migrated cells adhered to the bottom of the transwell membrane revealed a significantly higher number of THP-1 monocytes in the presence of heterotypic spheroids, with values of 4.5 × 10^− 7^ ± 2.85 × 10^− 7^cells / µm^2^ in homotypic spheroids and of 1.14 × 10^− 6^ ± 4.6 × 10^− 7^ cells / µm^2^ on the heterotypic ones, indicating an enhanced migratory capability when microglia is present (Fig. 7B and C). Chemically differentiated macrophages (M0), however, showed a similar response whether microglia was present on the co-culture or not, with values of 6.7 × 10^− 7^ ± 3.2 × 10^− 7^ cells / µm^2^ in cells cultured with CM of homotypic spheroids and of 6.5 × 10^− 7^ ± 1.5 × 10^− 7^ cells / µm^2^ for cells cultured in CM from heterotypic spheroids. This could be attributable to the pore size of the transwell membrane (8 μm) or the lower plasticity of the cells to deform and migrate through the pores. Nevertheless, spontaneous differentiation of THP-1 cells towards M2-like macrophages was increased in the presence of microglia, as previously shown (Fig. 6). Thereby, the presence of microglia demonstrated a pro-migratory impact within the heterotypic model on peripheral blood monocytes.

### Pro-tumoral secretome profile of heterotypic spheroid models

**Fig. 8:**
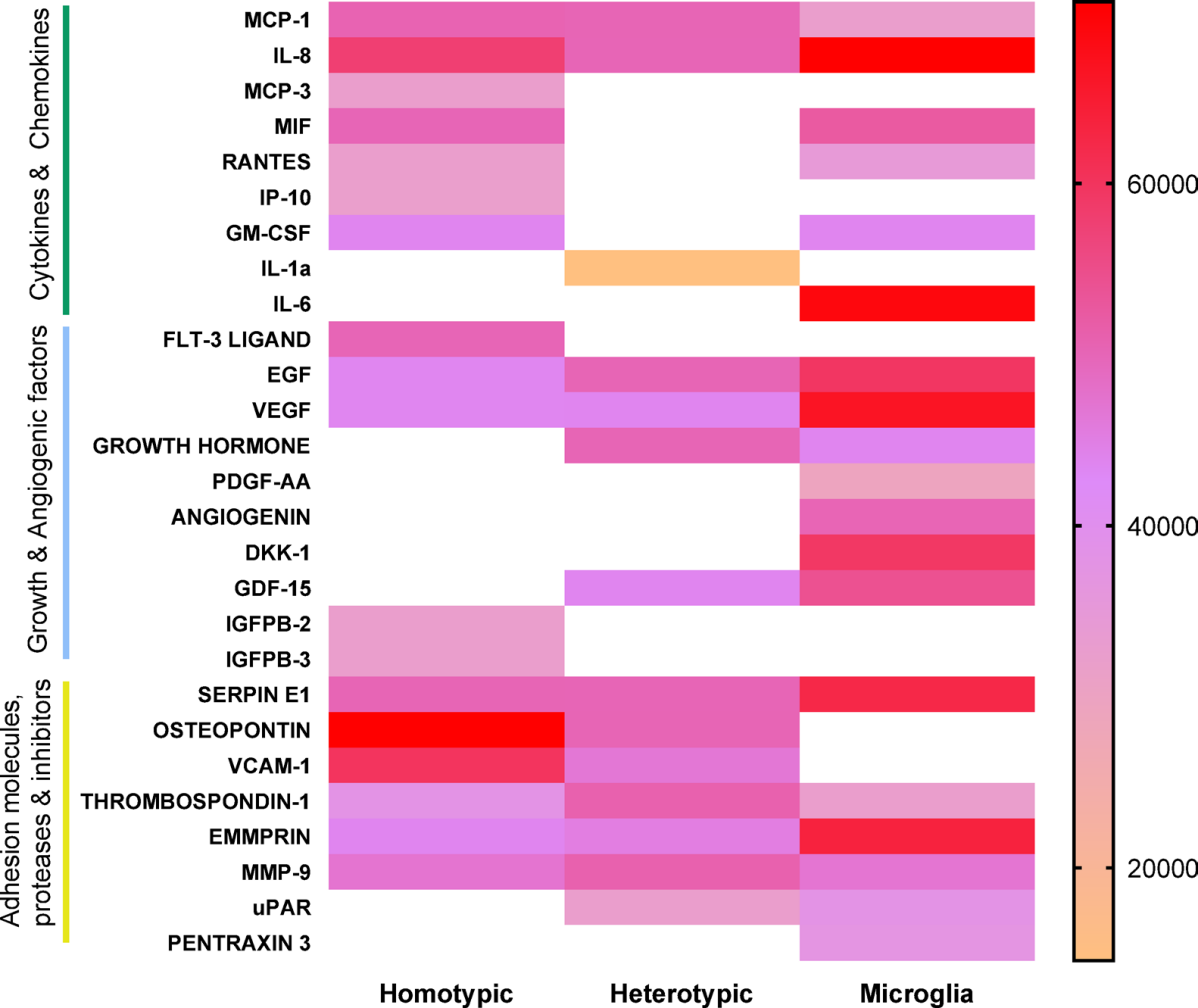
Secretome profile of homotypic and heterotypic spheroids. Heatmap of the relative protein secretion levels across the three spheroid conditions (homotypic and heterotypic GB22-13-based spheroids and homotypic microglia spheroids). Data is presented as the mean pixel intensity of technical duplicates. White spaces represent secretion below the detection limit.

To further understand the influence of microglia within heterotypic spheroids on modulating the tumor response and on the TME, the secretome was analyzed via a multi-array cytokine secretion analysis. To do so, the secretome of GB22-13 homotypic and heterotypic spheroids and microglia homotypic spheroids was collected after 5 days of culture. The diverse range of secreted factors was categorized into three primary clusters; (i) cytokines and chemokines, (ii) growth factors and angiogenic factors, and (iii) adhesion molecules, proteases, and inhibitors (Fig. 8). Analysis of the secretome demonstrated clear differences across all three experimental conditions, highlighting the unique role of microglia on the heterotypic spheroids.

Homotypic spheroids exhibited a secretome characterized by elevated concentrations of several pro-inflammatory and pro-angiogenic factors. Specifically, high expression levels of macrophage chemoattractant protein-1 (MCP-1), interleukin-8 (IL-8), macrophage migration inhibitory factor (MIF), Flt-3 ligand, osteopontin (OPN), vascular cell adhesion molecule-1 (VCAM-1), thrombospondin-1 (TSP1), and matrix metalloproteinase-9 (MMP-9) were observed. Moreover, the secretome of homotypic spheroids also presented elevated concentrations of monocyte chemoattractant protein-3 (MCP-3), FLT-3-Ligand and IGF-binding protein-2 and − 3 (IGFPB-2 and − 3), not present in other conditions. These markers have been related to characteristics such as high cell proliferation and recruitment of dendritic cells and infiltration of TAMs^30,41–45^, representing a pro-inflammatory and anti-tumoral environment.

Heterotypic spheroids presented a general decrease in overall cytokine release as compared to homotypic spheroids, which could be ascribed to the lower cell number per cell type (although the total cell number was kept constant). This co-culture condition also induced a shift in the cytokine profile. Interleukin-1 alpha (IL-1α), associated to migration and invasion of glioma cells, was uniquely expressed in this condition^61,62^. Additionally, an increased expression of MCP-1, IL-8, growth hormone (GH), growth differentiation factor 15 (GDF-15), osteopontin, VCAM-1, TSP-1, MMP-9, and urokinase plasminogen activator receptor (uPAR) was measured, markers associated with tumor malignancy, migration and invasiveness.

Microglia spheroids demonstrated a distinct cytokine secretion profile. A unique expression of interleukin-6 (IL-6), platelet-derived growth factor-AA (PDGF-AA), angiogenin (ANG), and Dickkopf-1 (DKK-1) was detected. Furthermore, increased levels of IL-8, vascular endothelial growth factor (VEGF), and extracellular matrix metalloproteinase inducer (EMMPRIN) were also recorded. These findings highlight the substantial influence of microglia on the secretome of glioma when co-cultured, evidenced by the unique cytokine profiles and altered expression patterns that contribute to the characteristic malignant behavior of GBM.

## Discussion

3D spheroid models of glioblastoma have been developed in the literature as a way to overcome the problems in the drug development pipeline and to study the interplay between different cell types building up the complex GBM microenvironment. One key player in GBM tumor development and aggressiveness is microglia cells. While some reports have demonstrated the impact of microglia on glioma malignancy and pro-tumorogenic response, replicating all hallmarks of GBM with in-vitro models is yet not possible. This is mainly because these models exploit cell lines such as U87, U251 and LN229, which are not able to recapitulate all key characteristics of GBM or simply because exhaustive analysis on these hallmarks is not conducted.

In order to study the impact of microglia in 3D in-vitro models of GBM and the impact on the use of different sources of glioma cells, heterotypic spheroids containing glioma and microglia cells, on a 70:30 ratio, were established from a commercially available cell line, DKMG, and from patient derived glioma stem cell line, GB22-13. GB22-13 cells, isolated from a tumor biopsy resection, are a mesenchymal subtype of GBM, clinically significant and directly correlated with a poor patient prognosis^24,25^.

Heterotypic spheroids provided clear insights of the importance of incorporating microglia. Heterotypic models consistently demonstrated enhanced proliferation with both DKMG and GB22-13 cells showing an 1,4 ± 0,3 and 1,3 ± 0,1 fold-increase in cell number after 7 days of culture, respectively (Figs. 1E and 2E). Similarly, previous studies on 2D co-culture models showed a 2.5-3,7 fold-increase in the proliferation of glioma cells in the presence of microglia^26^. In 3D, heterotypic spheroids of glioma and microglia have also demonstrated an increased proliferation, leading to significantly larger spheroids, ascribed to the upregulation of CCL18 in microglia cells, which in turn activates the CCR8 receptor in glioma cells, promoting glioma growth and invasion^27^.

GB22-13-based heterotypic spheroids formed multi-nucleated structures, a phenomenon particularly relevant given that GB22-13 cells originate from the mesenchymal cell subtype (Fig. 2F). The loss of sphericity given by the rising formation of multi-nucleated spheroids has been linked with malignancy in the GBM cell line U87^28,29^.

A slower invasion rate over time on an ECM-mimicking substrate was detected for GB22-13-based heterotypic spheroids (Fig. 3), a finding consistent with previous literature^30^. However, when heterotypic spheroids were prepared from DKMG cells, the invasion rate was enhanced in the presence of microglia, suggesting a differential interaction between microglia and DKMG or GB22-13 cells. Interestingly, the slower invasion rate observed in GB22-13-based heterotypic spheroids correlates with the previously described effect of microglia on tumor cell proliferation or migration, often termed the “go or grow” (migration/proliferation dichotomy) phenomenon^31,32^. By this phenomenon, microglia release factors that can either switch the glioma cell phenotype to a proliferative state (e.g., TGF-β) or to a migratory phenotype (e.g., MMP-9, EGF and VEGF). Thus, while we observe a higher proliferation in the presence of microglia cells, migration is reduced, suggesting a “grow” effect in our GB22-13-based heterotypic spheroids. On the contrary, DKMG-based heterotypic spheroids display both, increased proliferation and migration capability on the presence on microglia, which could be an artifact of the use of this particular cell line.

Analysis of the specific cells driving invasion using GFP-labeled GBM cells (Fig. 3H), revealed that in heterotypic spheroids, microglia primarily lead invasion at the spheroid borders. This observation reflects the localization of microglia in vivo, where they are predominantly observed within the peritumoral area and infiltrative margins of GBM tumors^33^. The invasion of microglia is likely led by hypoxic conditions generated in high-density areas, where cells tend to escape^33,34^. Notably, it has been shown that microglia tends to present a random walk with increased motility on the infiltrative margins whereas glioma presents a diffusion invasive pattern, although the mechanism behind this is still unknown^9^.

Heterotypic spheroids demonstrated an enhanced drug resistance mediated by the presence of microglia (Fig. 4). This finding aligns with previous studies that have shown that co-cultures of microglia and glioma cells result in a reduced sensitivity to TMZ, due to the upregulation of IRDS gene in glioma cells, directly linked to chemotherapy resistance^13,35^. Analysis of the percentage of glioma and microglia cells present after treatment with TMZ, showed a higher relative decrease of glioma cells as compared to microglia, which would support the idea of the microglia acting as a protective layer.

Detailed investigation of the cellular organization on heterotypic DKMG-based and GB22-13-based spheroids revealed a core-shell arrangement, where tumoral cells are protected by the microglia shell (Fig. 5). This result correlates with the presence of microglia on the adjacent invasive areas of the GBM tumors as demonstrated by using spatial transcriptomic analysis, where the GBM tumor has been divided in four layers with the outer two being predominately composed by microglia^22,36^. Regardless of these important cellular arrangements observed in native GBM tumors, to the best of our knowledge, it is the first time that such cellular organization has been reported in the literature for in-vitro spheroid models.

Consistent with existing literature, heterotypic spheroids demonstrated that the presence of microglia significantly alters the phenotype of monocytes, polarizing them to M2-like (anti-inflammatory) macrophages (Fig. 6). This was evidenced by the increased presence of adhered cells (macrophage differentiated monocytes) when non-adherent monocytes were cultured with CM from heterotypic spheroid cultures and, the expression of the M2 surface marker CD-163 on adhered cells, which is commonly upregulated in the context of GBM^37–39^. This phenotypic shift demonstrated an immunosuppressive microenvironment within our in vitro model, mirroring observations in GBM tissue explants where TAMs exhibit a similar anti-inflammatory profile that supports tumor growth^26^. Clinically, a high expression of CD163 is frequently observed in the surrounding brain parenchyma of GBM patients, and this elevated expression is associated with poorer survival rates^28,29^.

As discussed earlier, in-vitro models of GBM generally lack the ability to recreate all hallmarks of GBM, presumably due to the use of established cell lines. As evidence from our data, GB22-13 cells better recreated these features of GBM than the DKMG cell line, with a more realistic “go or grow” effect where migration or proliferation of tumoral cells is differentially activated, the formation of multinucleated structures (Fig. 2E), an increased drug resistance (Fig. 4D and E) and an increased monocyte differentiation (Fig. 6).

One of the most outstanding characteristic of GBM is the well-known cell recruitment capability. Tumoral cells are known to secrete a number of factors, such as IL-10, TGF-B and CSF-1 amongst others, that influence the immuno-modulatory response^40^. The heterotypic spheroids developed here were able to recruit monocytes on a transwell co-culture model, to a greater extent than the homotypic versions of these.

Analysis of the secretome of heterotypic, homotypic and microglia homotypic spheroids revealed distinct patterns amongst the three different culture conditions. The secretome of homotypic glioma spheroids was characterized by the unique presence of IGBFP-2/3, FLT-3, MCP-3, IP-10, GM-CSF and MIF proteins (Fig. 8). High levels of IGFBP-2/3, are related to high cell proliferation^30,41^, while Ms-like tyrosine kinase 3 ligand (FLT-3 ligand) mediates the recruitment of dendritic cells, harnessing an anti-tumoral response^42–44^. Okada et al.^45^ demonstrated that monocyte chemotactic protein 3 (MCP-3) mediates the infiltration of TAMs in GBM. Macrophage migration inhibitory factor (MIF) works as a pro-angiogenic factor with high expression levels being found in GBM in patients with malignant tumors^46,47^. Interferon gamma-induced protein (IP-10/CXCL10) is known to recruit T cells towards the tumoral zone. The granulocyte-macrophage colony-stimulating factor (GM-Csf), also synthetized by homotypic glioma spheroids, is involved in the pro-inflammatory response by promoting IL-4 expression^49,50^, but has been shown to also promote cancer growth, favoring tumor progression^51^.

The secretome of homotypic microglia spheroids showed the unique synthesis of IL-6, PDGF-AA, ANGIOGENIN and DKK-1. IL-6 plays a neuroprotective and regenerative role in the homeostasis of the brain^52^. High levels of the vascular endothelial growth factor (VEGF) on microglia cultures may work as a stimulus for monocyte migration and recruitment^53,54^. Platelet-derived growth factors homodimers (PDGF-AA) is also known to promote tumor growth, angiogenesis and drug resistance^55,56^. Dickkopf1 (DKK-1), when found at high levels results in an aberrant activation of the Wnt signaling and can promote tumor metastasis^57,58^. EMMPRIM is known to stimulate stromal cells to produce MMPs, enhancing tumor invasion and proliferation^59,60^.

Heterotypic spheroids shared key secretome proteins with both, homotypic glioma and microglia spheroids, even though the presence of microglia was only of 30%. Higher malignancy factors were found in heterotypic spheroids. Here, interleukin-1 (Il-1), generally associated with migration and invasion in the mesenchymal subtype of glioma cells was uniquely expressed^61,62^, which is in agreement with the results found in higher cell proliferation on heterotypic spheroids (Fig. 1). Macrophage inhibitory cytokine-1 (GDF-15) serves as a biomarker for malignant cancer progression, high concentrations of this protein is related with lower survival^63^, and it was found on the secretome of both, microglia and heterotypic spheroids. SERPIN E1 is essential for GBM development, a high expression is correlated with aggressiveness, poor prognosis and resistance to TMZ^64–66^, which was highly synthetized by homotypic microglia and heterotypic spheroids and reflects the higher cell viability observed on heterotypic spheroids after treatment with TMZ (Fig. 4).

Matrix metalloproteinase-9 (MMP-9) is essential for GBM progression, by degrading the brain ECM and enabling an easier penetration and movement of tumoral cells. In fact, a high expression of MMP-9 is characteristic of a pro-invasive phenotype, proving the malignancy of the tumor^36,67,68^. This protease was synthesized by heterotypic spheroids, and in a higher level than the homotypic glioma and microglia counterparts. Similarly, osteopontin (OPN) is expressed in the GBM mesenchymal lineage and involved in invasion and radiation resistance, appeared also highly present on the secretome of heterotypic spheroids^69,70^. And, lastly, induced vascular cell adhesion molecule-1 (VCAM1), present in microglia and heterotypic spheroids, is a protein highly expressed on high invasive malignant tumor margins^71^. Surprisingly and despite the presence of MMP-9, OPN and VCAM1, our invasion studies showed a reduced invasion in heterotypic spheroids (Fig. 7). Notably, the urokinase receptor (uPAR) that promotes GBM survival, migration and serves as a biomarker correlated to the high survival of the mesenchymal subtype, especially under hypoxic conditions, was highly present in the heterotypic spheroids which could be related to the high viability found within the heterotypic GB22-13 spheroids (Fig. 2B)^72–74^.

In summary, our studies on the development of heterotypic microglia containing GBM spheroids demonstrated that these models hit multiple hallmarks of the native GBM, presenting a unique platform for drug screening and the study of glioma-glia interactions. Heterotypic spheroids demonstrated a higher proliferative character with the formation of multinucleated structures, promoted the recruitment of monocytes and their differentiation towards M2-like macrophages suggesting potential for activation of the immune system towards a pro-tumoral environment and presented and increased drug resistance. Moreover, cells within heterotypic spheroids presented a spatial arrangement that to the best of our knowledge has only been observed in-vivo, highlighting the significance of the developed model system. While our findings illuminate the protective role of this microglia-rich shell, further studies are essential to fully elucidate its precise impact and establish its direct correlation to the border regions observed in vivo^22^. This validation would significantly enhance the physiological relevance of our in vitro models, improving their capacity to mimic the patient-specific tumor microenvironment. Additionally, further investigation would be needed to determine if the presence of multinucleated spheroids is a characteristic specific to the mesenchymal lineage of patient-derived glioma cells, which could provide deeper insights into lineage-specific tumor behaviors and treatment responses.

## Supplementary Information

Below is the link to the electronic supplementary material.

Supplementary Material 1

## Data Availability

The datasets generated during and/or analysed during the current study are available from the corresponding author on reasonable request.
