# Supplementary material for: 3D heterotypic models of glioblastoma reveal the impact of microglia on cellular organization and the production of a distinct secretome
Source: Sci Rep. 2026 Feb 4;16:7246. doi: 10.1038/s41598-026-37395-0 (PMC12923614; doi:10.1038/s41598-026-37395-0)
Supplement: Supplementary file 1 — Supplementary Material 1 [file 41598_2026_37395_MOESM1_ESM.pdf]

3D heterotypic models of glioblastoma reveal the impact of microglia on cellular organization and the production of a distinct secretome.

Clara García-Sáez[1], Josune Alonso-Marañón[2], Mikel García-Puga[2], Ane Rubio-Zulaika[2], Irati de Goñi-Garcia[3], Lorea Blázquez[2,4,5] and Sandra Camarero-Espinosa[1,4*]

[1]BioSmarTE Lab, POLYMAT, University of Basque Country UPV/EHU, Av. de Tolosa, 72, 20018, Donostia-San Sebastián, Spain.

[2]Neurosciences Area, Biogipuzkoa Health Research Institute, 20014 San Sebastian, Spain

[3]Neurosurgery department, Donostia University Hospital, 20014 San Sebastian, Spain

[4]Ikerbasque, Basque Foundation for Science, Euskadi Pl., 5, 48009, Bilbao, Spain.

[5]Centro de Investigación Biomédica en Red de Enfermedades Neurodegenerativas (CIBERNED), Instituto de Salud Carlos III, Madrid, Spain.

*Corresponding author: Sandra Camarero-Espinosa

E-mail: sandra.camarero@ehu.eus

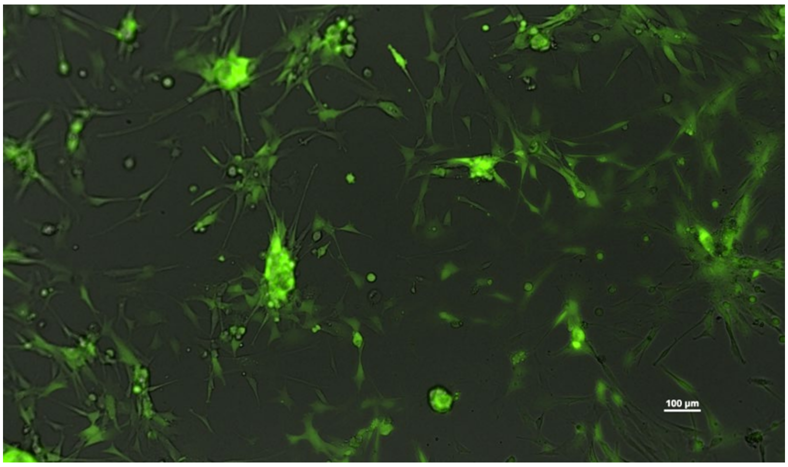

Figure S1. Fluorescence microscopy image of GB22-13 cells after transduction with lentiviral (Lv) green fluorescent protein (GFP) vector and selection. The scale bar is 100 µm.

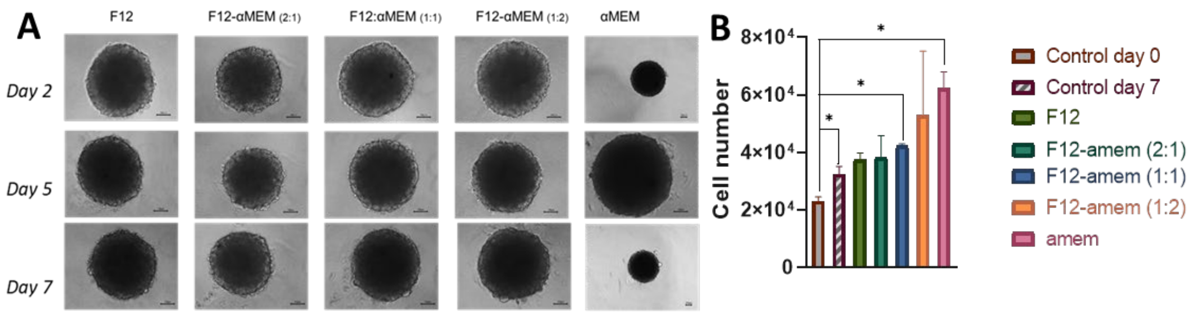

Figure S2. (A) Optical microscopy images of DKMG heterotypic spheroids after culture for 2, 5 and 7 days with different media compositions (DMEM/F12 supplemented neurobasal media to α-MEM, with ratios of 1, 2:1 , 1:1 , 1:2 and 1). **(B)** Cell number as calculated from total DNA quantification for DKMG heterotypic spheroids under the distinct media compositions. Statistical significances of spheroids (n=3) measurements were calculated from two-way ANOVA with posthoc Tukey's multiple comparison tests; (****) p<0.0001, (***) p<0.001, (**) p<0.01, and (*) p<0.1 was used.

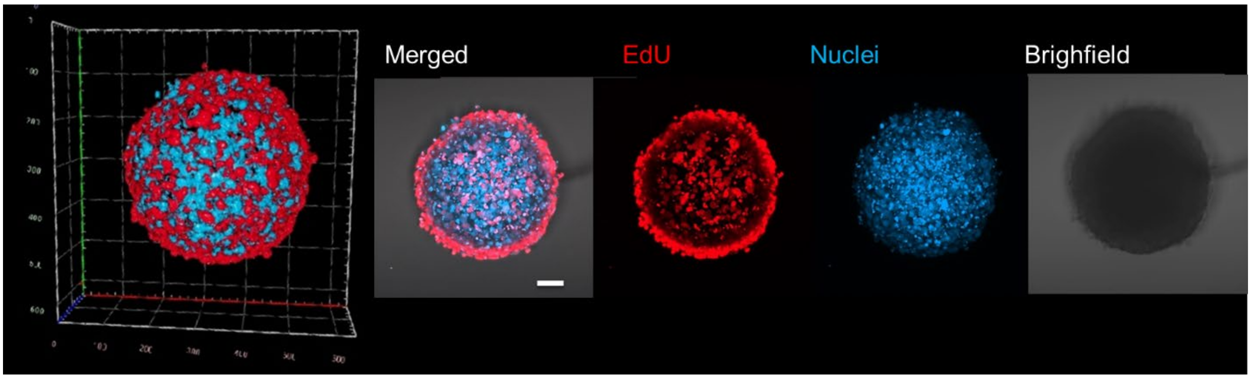

Figure S3. EdU incorporation in a heterotypic spheroid. A confocal image of a DKMG heterotypic spheroid after 7 days of culture. Proliferating cells are labeled with EdU (red), and nuclei are counterstained in blue. The scale bar is 100 µm.

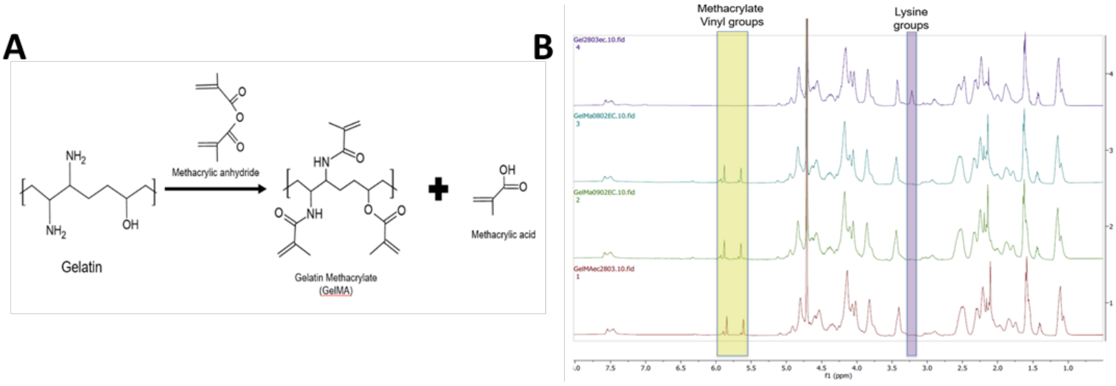

Figure S4. (A) Scheme of the reaction for the metacrylation of gelatin. **(B)** ^{1}H-NMR analysis showing the characteristic methacrylic groups (yellow) visible as double-doublets at 5.7–5.9 ppm. The spectra were normalized to the lysine groups (violet) within the gelatin backbone to ensure accurate quantification.

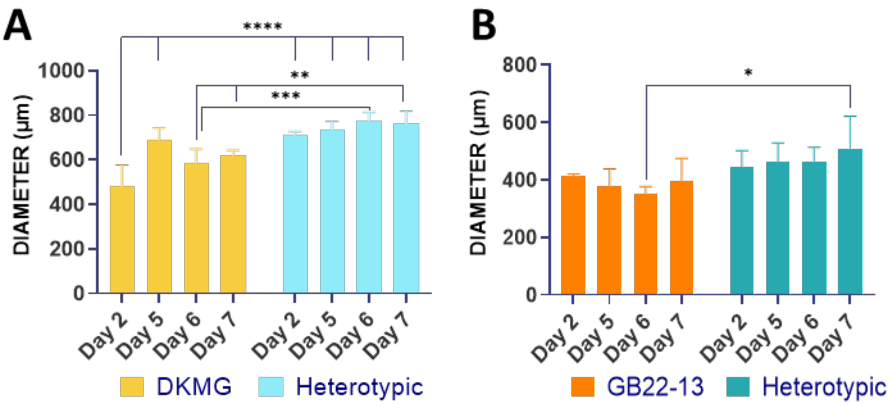

Figure S5. Diameter of DKMG-based **(A)** and GB22-13-based **(B)** homotypic and heterotypic spheroids over culture time. The data was collected on days 2, 5, 6, and 7 (n=5). Statistical significance was calculated with a two-way ANOVA with posthoc Tukey's multiple comparison tests; (****) p<0.0001, (***) p<0.001, (**) p<0.01, and (*) p<0.1. n = 5

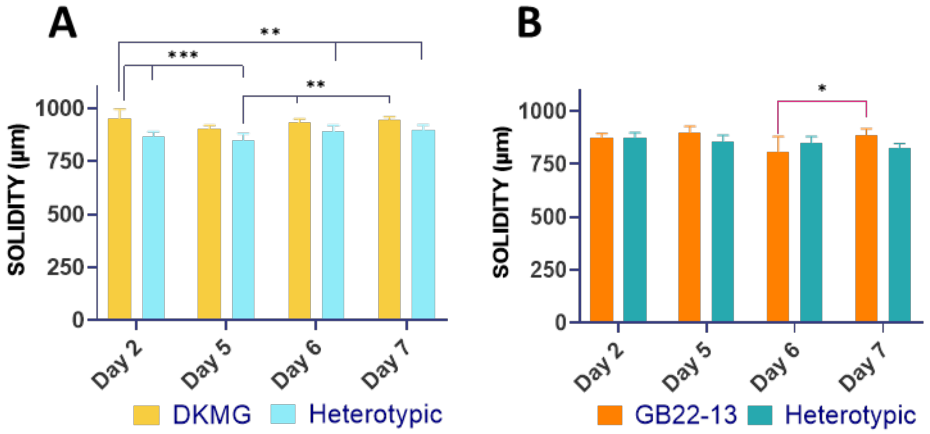

Figure S6. Solidity of DKMG-based **(A)** and GB22-13-based **(B)** homotypic and heterotypic spheroids over time. Data was collected on days 2, 5, 6, and 7, with each data point representing the average of five replicates (n=5). Statistical significances was calculated from n = 5 spheroids with a two-way ANOVA with posthoc Tukey's multiple comparison tests; (****) $p<0.0001$, (***) $p<0.001$, (**) $p<0.01$, and (*) $p<0.1$.

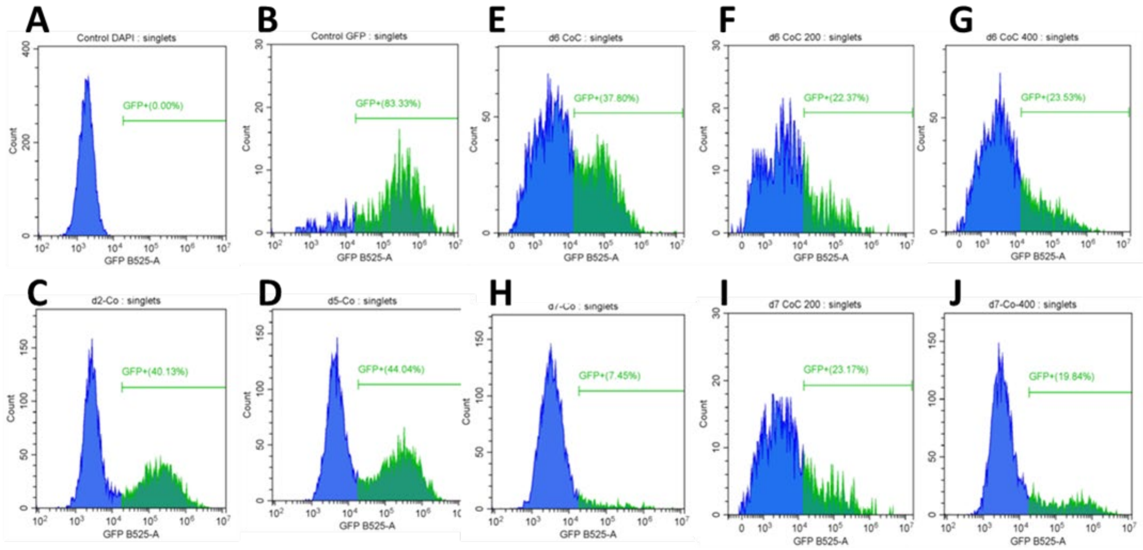

Figure S7. Flow cytometry histograms showing the cell count of Hoescht+ and Hoesch+/GFP+ cells in samples of **(A)** HMC3 cells stained for Hoescht, **(B)** GFP-GB22-13, cell populations 2 **(C)** and 5 days **(D)** after culture of spheroids. The effects of temozolomide (TMZ) treatment are shown in panels (**E-F-G)** after 24h of treatment with 0 µM, 200 µM and 400 µM TMZ, respectively. Panels (**H-I-J)** present the cell populations after 48 h of treatment with TMZ at concentrations of 0 µM, 200 µM and 400 µM TMZ, respectively

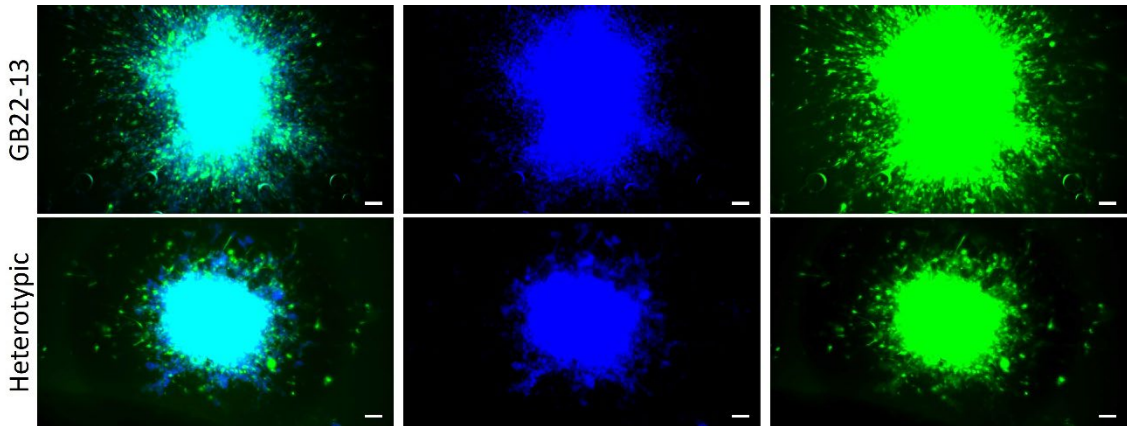

Figure S8. Fluorescence microscopy images of homotypic and heterotypic spheroids on top of the GelMA films using GFP-GB22-13 cells (green) and counterstained for DNA (all cells, Hoechst, blue). Scale bar is 100µm for all images.

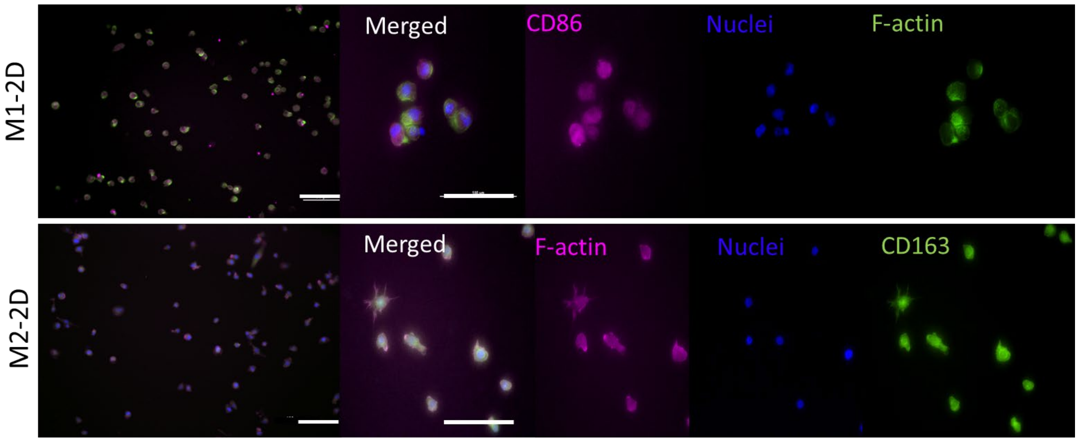

Figure S9. THP-1 macrophage polarization. Fluorescence microscopy images of THP-1 macrophages after chemical polarization to M1 (top row) or M2 (bottom row) phenotype. M1 cells are positive for CD86 (pink), while M2 cells are positive for CD163 (green). In both panels, nuclei are stained blue, and F-actin is stained for morphology (green in the top panel; pink in the bottom panel). Scale bar is 100 µm for all images.
